# Forefront Strategies for Biomass Valorization: From Deconstruction to Bioplastic Production

**DOI:** 10.1021/acsmaterialsau.5c00023

**Published:** 2025-06-02

**Authors:** Jaya Baranwal, Danila Merino

**Affiliations:** † POLYMAT, 652638 University of the Basque Country UPV/EHU, Joxe Mari Korta Center, Avda. Tolosa 72, 20018 Donostia-San Sebastian, Spain; ‡ Ikerbasque, Basque Foundation for Science, 48009 Bilbao, Spain

**Keywords:** Agro-waste valorization, Bioplastics, Biomass deconstruction, Ionic solvents, Green solvents, Deep eutectic solvents, Renewable resource, Circular economy, Thermomechanical processing

## Abstract

This Review highlights cutting-edge strategies for transforming agricultural residues into bioplastics, offering a sustainable alternative to conventional petroleum-based plastics. By focusing on the deconstruction and reassembly of nonedible agro-wastes, these methods address critical challenges such as resource competition, plastic pollution, and greenhouse gas emissions. Key techniques reviewed include biomass dissolution, hydrolysis, and thermomechanical processing, with particular emphasis on the use of greener solvents such as ionic liquids (ILs) and deep eutectic solvents (DES). These approaches demonstrate significant potential for minimizing waste, improving resource efficiency, and enabling circularity in bioplastic production. The Review also critically examines current limitations, including solvent toxicity, scalability, and economic feasibility, while identifying promising directions for future research. By integrating innovative deconstruction techniques with sustainable manufacturing practices, this work aims to unlock the full potential of agricultural residues, paving the way toward a zero-waste, biobased economy.

## Graphical abstract

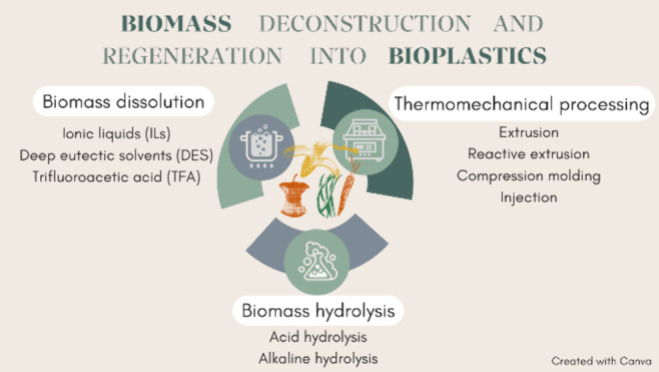

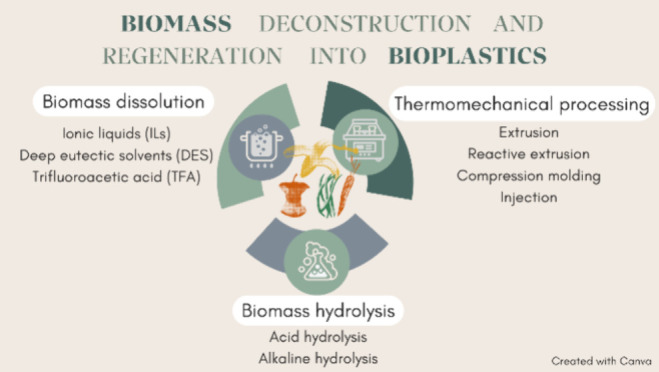

## 1 Introduction

Plastics are ubiquitous in modern life and are valued for their versatility, durability, and cost-effectiveness. However, their production—largely dependent on fossil fuels—has severe environmental consequences, including significant carbon emissions and plastic pollution.^1^ Since the 1950s, global plastic production has grown exponentially, reaching 400.3 million tons in 2022, with nearly half of these products designed for single-use applications.^2–4^ Discarded plastics, resistant to degradation, persist in the environment for centuries, contributing to the accumulation of approximately 5 billion tons of waste to date.^3^

This persistence gives rise to micro- and nanoplastic particles, now recognized as emerging pollutants with far-reaching impacts on terrestrial and aquatic ecosystems.^5^ These particles infiltrate food chains, posing risks to plant and animal health and ultimately threatening human well-being.^6–8^ Efforts to mitigate these issues—such as recycling and incineration—face significant technical and economic challenges. Recycling is limited by the degradation of polymer quality during reprocessing, while incineration, despite enabling energy recovery, releases greenhouse gases that exacerbate climate change.^9–11^

A transformative shift in plastic production is urgently needed, one that prioritizes renewable resources, biodegradability, and circularity. Bioplastics, produced from renewable sources, biodegradable, or both, present a promising alternative to fossil-based plastics.^12^ Nonedible agricultural residues and food industry byproducts are particularly attractive feedstocks due to their abundance, sustainability, and rich composition of cellulose, hemicellulose, lignin, and proteins.^13,14^ By valorizing these waste materials, bioplastics not only reduce reliance on fossil fuels but also support waste management strategies in line with a circular economy.^15^

This Review explores cutting-edge methods for converting plant biomass into bioplastics. Traditional approaches—such as polymer extraction from biomass,^16,17^ polymer production through natural or genetically modified microorganisms,^18,19^ and their synthesis from biobased monomers^20,21^ —have shown promise but remain limited by high costs, complex processing steps, and waste generation. In contrast, emerging strategies focus on the deconstruction and reassembly of raw materials into biocomposites with tailored properties. This article highlights three key approaches: (1) direct dissolution of biomass in innovative solvents, (2) controlled chemical transformation through hydrolysis, and (3) physicochemical modification via thermomechanical processes.

By critically evaluating the advantages, limitations, and future potential of these methods, this Review aims to advance the development of sustainable bioplastic technologies. These innovations represent a critical step toward achieving a biobased circular economy and addressing the global challenges posed by plastic waste and climate change.

## 2 Chemical Interactions in Plant Biomass

The potential of plant biomass for bioplastic production lies in its structural and compositional complexity, particularly in the intricate architecture of the plant cell walls. These walls, which constitute up to 90% of plant cell dry matter, are natural nanostructured networks of biopolymers.^22,23^ Their composition and interactions underpin the recalcitrance of biomass, posing challenges for efficient deconstruction but also offering opportunities for tailored valorization processes.

Plant cell walls are typically categorized into primary and secondary walls (Figure 1), each serving distinct structural and functional roles. Primary walls that support growth are composed of cellulose, hemicellulose, and pectin. Pectin-rich biomass, such as citrus peels and fruit pomace, contains between 12 and 35% pectin and minimal lignin content, making it more suitable for direct valorization into bioplastics.^24,25^ In contrast, secondary walls, which develop in response to mechanical stress, are enriched in lignin and contribute to the rigidity and hydrophobicity of lignocellulosic biomass, such as agricultural residues and forestry byproducts. These materials typically contain 30–50% cellulose, 20–40% hemicellulose, and 10–35% lignin.^26,27^

**Figure 1:**
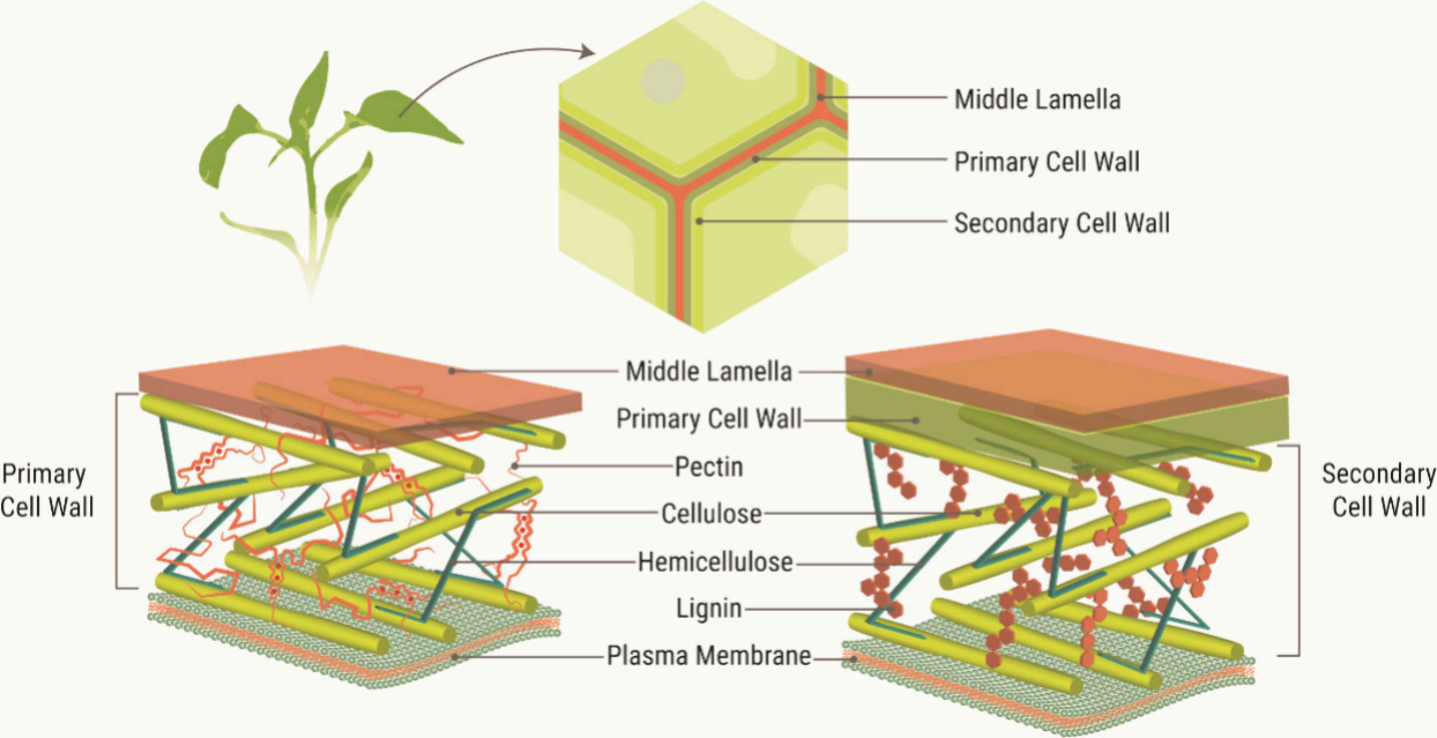
Visual representation of the structure and composition of plant cell walls. Adapted with permission under a Creative Commons, Attribution 3.0 Unported License, from Merino et al., 2022.^14^ Copyright 2022 Royal Society of Chemistry.

### 2.1 Cellulose

Cellulose is the most abundant polymer in plant cell walls, consisting of linear chains of β-1,4-linked d-glucose units. It forms a hierarchical structure, where strong intra- and intermolecular hydrogen bonding drives the assembly of microfibrils, which in turn aggregate into fibers (Figure 2). This results in crystalline regions that confer rigidity and hydrophobicity, as well as amorphous regions that are more reactive and accessible to chemical or enzymatic modification.^28,29^ The crystallinity index, which varies across biomass types, is a key determinant of cellulose’s reactivity. Higher crystallinity, as found in woody biomass, contributes to greater recalcitrance.^23^

**Figure 2:**
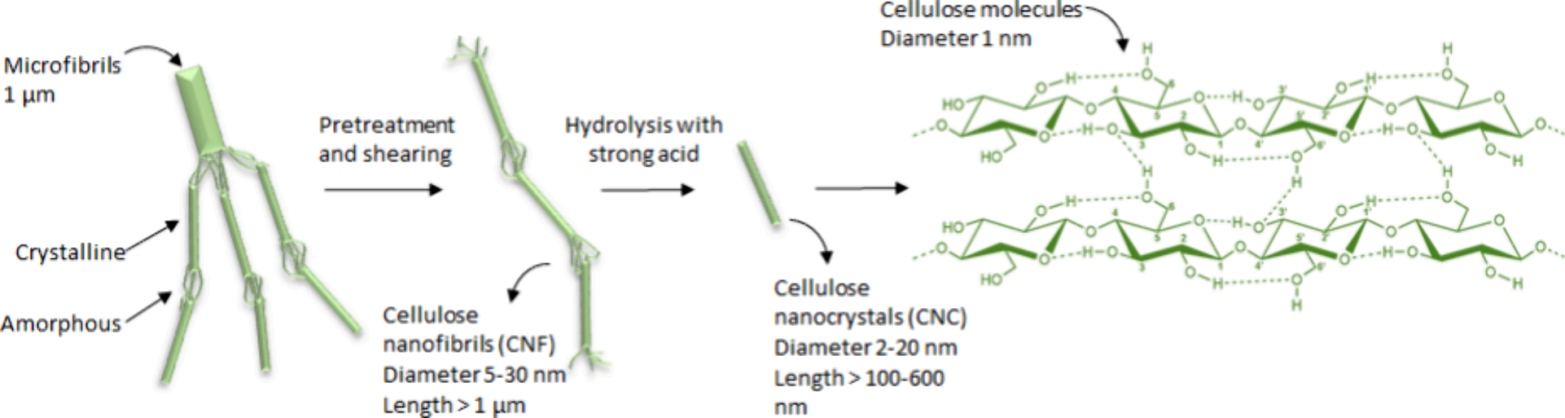
Supramolecular assembly of cellulose molecules driven by strong intra- and intermolecular hydrogen bonding (depicted by red dashed lines).

The strong hydrogen bonding network within cellulose microfibrils contributes to their resistance to dissolution. Disrupting these bonds through solvent-based approaches, such as ionic liquids (ILs) or deep eutectic solvents (DES), is a critical step in its valorization. Moreover, the amorphous regions of cellulose, being more susceptible to chemical attack, provide opportunities for selective functionalization, enabling the production of bioplastics with tunable properties.

### 2.2 Hemicellulose

Hemicellulose, a heterogeneous group of branched polysaccharides, plays a pivotal role in cross-linking cellulose microfibrils and lignin.^22,26,28^ Its structural diversity arises from variations in sugar monomers, such as xylose, mannose, glucose, galactose, and arabinose, as well as from the degree and type of branching (Figure 3). The most common types of hemicellulose include xylans, mannans, and glucomannans, each differing in composition and abundance depending on the biomass source.^30^

**Figure 3:**
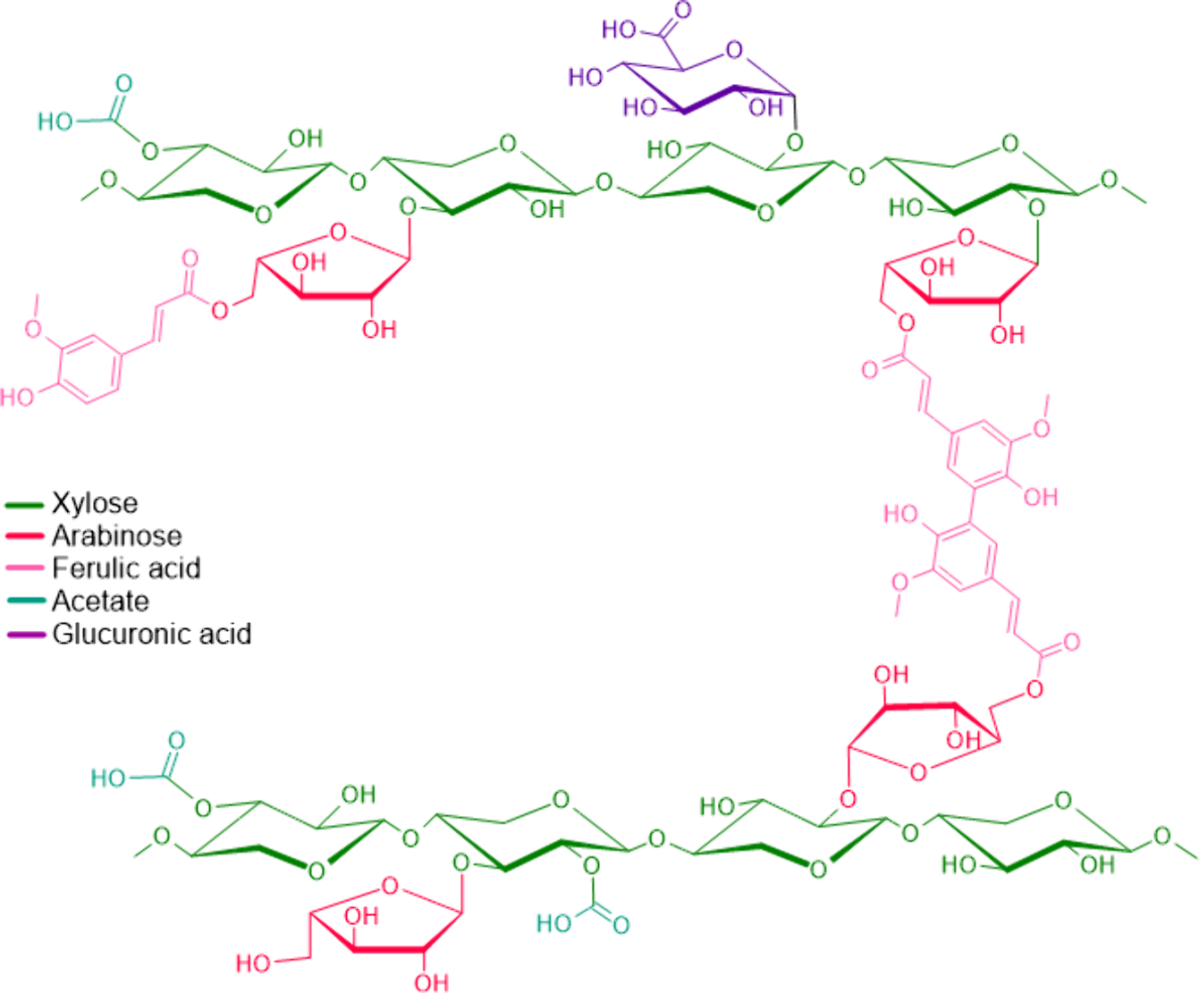
Chemical structure of xylan, representing a typical example of hemicellulose composition.

For example, xylans, which dominate in hardwoods, often bear acetyl groups and side chains, such as arabinose or glucuronic acid. These substitutions enable covalent and hydrogen bonding interactions with lignin, reinforcing the cell wall matrix.^30^ Similarly, glucomannans, abundant in softwoods, are partially acetylated, enhancing their solubility and reactivity. Hemicellulose’s amorphous and low-crystallinity structure makes it highly amenable to chemical and enzymatic deconstruction, providing a valuable feedstock for bioplastic production.^23^

### 2.3 Pectin

Pectin, primarily found in primary cell walls, fills the spaces between cellulose microfibrils and contributes to the wall porosity and flexibility. Its backbone of α-(1,4)-linked galacturonic acids forms domains such as homogalacturonan (HG), rhamnogalacturonan I (RG-I), and rhamnogalacturonan II (RG-II). HG domains, which are predominantly linear (Figure 4), play a crucial role in forming calcium-mediated cross-links, while the branched RG-I and RG-II domains contribute to the structural diversity of the cell wall.^22^

**Figure 4:**
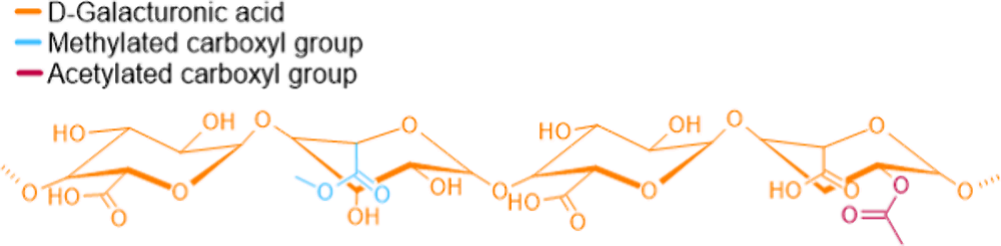
Chemical structure of homogalacturonan (HG), the linear and predominant domain found in pectins.

### 2.4 Lignin

Lignin, the dominant polymer in secondary cell walls, is a complex, aromatic macromolecule composed of *p*-hydroxyphenyl (H), guaiacyl (G), and syringyl (S) units (Figure 5A). These monomers are interconnected through various bonds, including ether and carbon–carbon linkages (Figure 5B), forming a three-dimensional network that provides structural rigidity and water resistance to the cell wall.^31^

**Figure 5:**
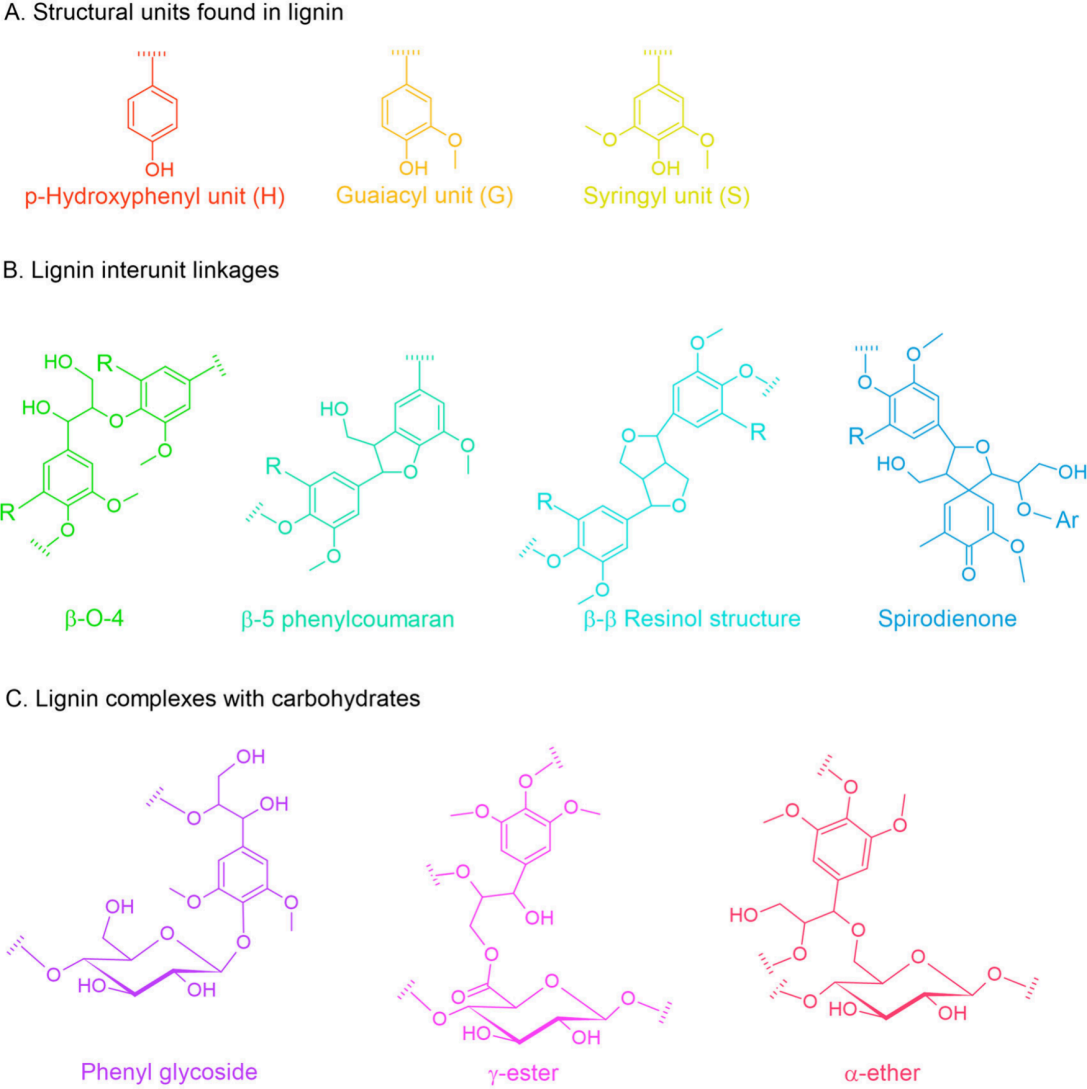
Structural features of lignin: (A) Lignin structural units, (B) key interunit linkages, and (C) lignin–carbohydrate complexes.

Lignin’s strong association with cellulose and hemicellulose through lignin–carbohydrate complexes (LCCs) contributes to biomass recalcitrance (Figure 5C). While lignin’s structural complexity poses challenges for its valorization, its aromatic nature offers unique opportunities for creating value-added biopolymers and bioplastics. Lignin’s insolubility in water and its structural complexity make it challenging to process, although specific solvents like alkaline solutions and organosolv technologies can cleave lignin bonds, enabling its modification for polymer applications.^23^

### 2.5 Proteins, Inorganic Compounds, and Lipids

Proteins, lipids, and inorganic compounds are minor but important components of plant biomass, typically incorporated within or as part of cell wall structures. Proteins are dispersed in the polysaccharide matrix and sometimes covalently bonded to cell wall molecules. Their side chain amino acids have reactive functional groups (e.g., carboxyl, hydroxyl, amine), which present the potential for cross-linking within polymer matrices.^32^ Lipids, waxes, fatty acids, and sterols are mainly stored in the cuticle and membrane matrices, playing roles in hydrophobicity and as barriers to water and microbial invasion.^33^ Inorganic compounds, mainly silica, calcium, potassium, and phosphorus, are deposited as salts or nanocrystals within cell wall surfaces or within cell walls.^34^

### 2.6 Recalcitrance and Biomass Valorization

The interplay of cellulose, hemicellulose, pectin, and lignin results in a highly recalcitrant material, with cellulose crystallinity, lignin content, and polymer interactions being major barriers to efficient processing. Overcoming this recalcitrance is essential for the effective valorization of biomass into bioplastics. Advanced deconstruction strategies, such as solvent dissolution, hydrolysis, and thermomechanical processing, aim to selectively disrupt these interactions while preserving or enhancing the functional properties of the biopolymers.

## 3 Strategies for Biomass Deconstruction and Reassembly into Bioplastics

### 3.1 Biomass Dissolution

Dissolving plant biomass effectively is a critical step in its transformation into bioplastics. The process requires carefully selected solvents capable of breaking the complex interactions between cell wall polymers. Recent advancements have identified ILs,^35^ DES,^36^ and trifluoroacetic acid (TFA)^37^ as promising candidates for targeted dissolution. This section examines cutting-edge strategies to leverage the full potential of biomass dissolution and regeneration with the aim to foster sustainable and efficient bioplastic production.

#### 3.1.1 Ionic Liquids

Plant biomass deconstruction is challenging due to its inherent recalcitrance, which stems from strong intermolecular interactions among macromolecules.^16^ Dissolution-based transformation offers a forward-thinking solution, enabling direct conversion of biomass into functional materials with minimal waste, low carbon footprints, and contributions to the circular economy.^38^

ILs, organic salts liquid at room temperature, have gained attention since Swatloski et al. first reported their use for cellulose dissolution in 2002.^39^ Capable of dissolving a wide range of biopolymers, ILs are particularly promising for processing lignocellulosic biomass. However, challenges such as cellulose’s dense hydrogen-bonding network, the complexity of biomass structures, and the need for high temperatures (175–185 °C) complicate the dissolution process.^38,40–42^ Pretreatments like ball milling or autohydrolysis are often required to improve solubility. Despite these challenges, ILs remain a focal point in research due to their versatility, performance, and recyclability (Figure 6).

**Figure 6:**
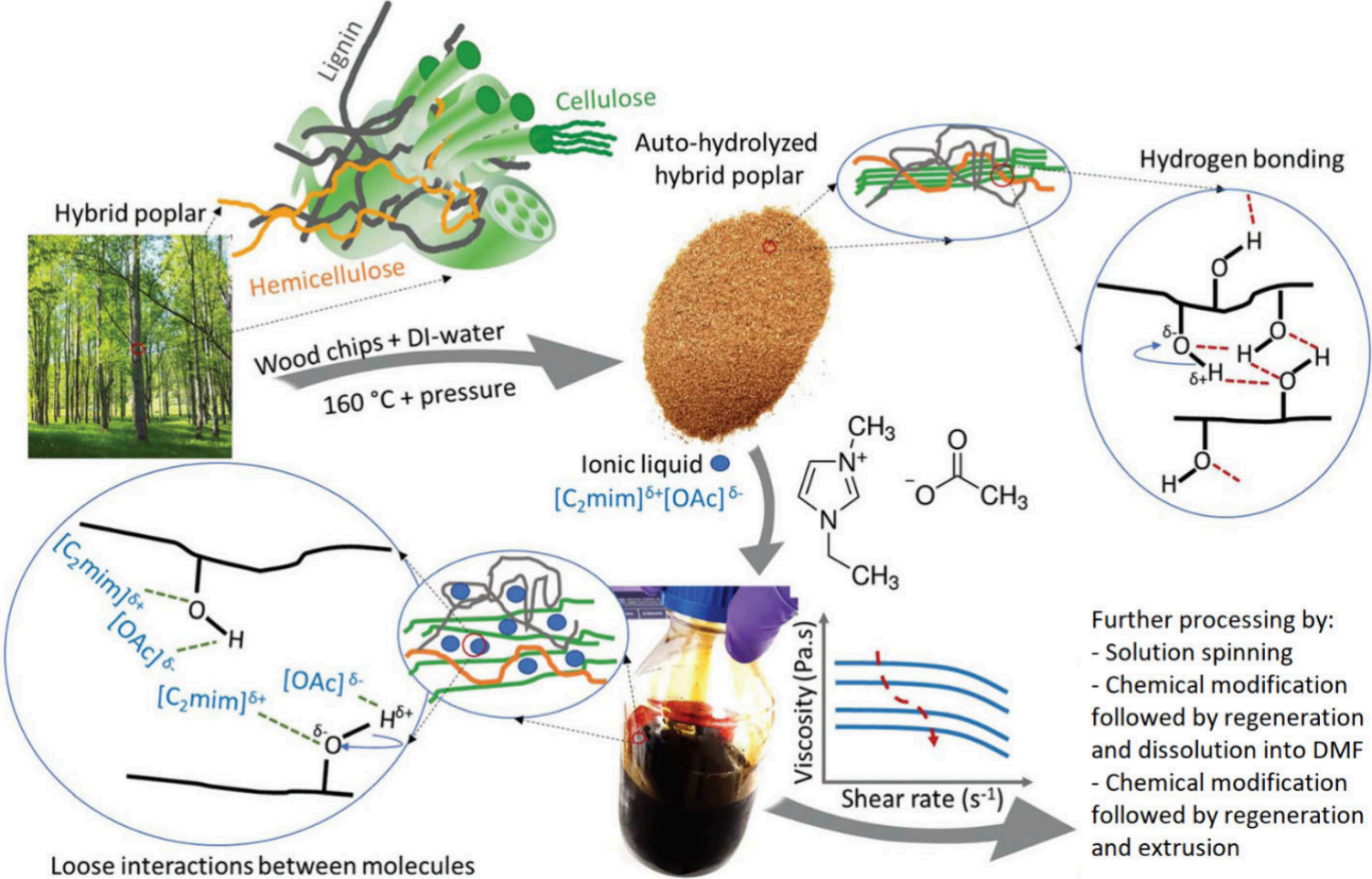
Representation of the process of dissolution of hybrid poplar biomass with 1-ethyl-3-methylimidazolium acetate ([EMIM]­[OAc]), ionic liquid, and some examples of possible processing routes after biomass dissolution. Reproduced with permission from from Nguyen et al.^42^ Copyright 2025 Royal Society of Chemistry.

According to the literature, cellulose can be dissolved up to 30 wt % in 1-allyl-3-methylimidazolium chloride ([AMIM]­[Cl]),^43^ up to 25 wt % in 1-butyl-3-methylimidazolium chloride ([BMIM]­[Cl]) using microwave heating,^39^ and up to 16 wt % in 1-ethyl-3-methylimidazolium acetate ([EMIM]­[OAc]) heating at 80 °C.^44^ In all cases, cellulose dissolution is achieved thanks to the interactions between the hydroxyl groups of cellulose with the anions and cations of the IL, which causes the breakage of the hydrogen bonding between the molecular chains of cellulose.^35^ Because of this, these ILs have been used for biomass dissolution and derivatization. In general, the use of ILs for the preparation of bioplastics involves a two-step process in which the biomass is first dissolved in the ILs, and second, it is chemically modified before its precipitation by the addition of an antisolvent. After that, the modified biomass can redissolve in an organic solvent and the bioplastics can be obtained by solvent casting^45^ or injection molding.^46–48^ Examples are listed in Table 1.

**Table 1:** Summary of the Latest Published Works Reporting on the Dissolution of Biomass in ILs for the Preparation of Bioplastics

Vegetable waste	Composition	Biomass Pretreatment	Solvent	Procedure	Main results	References
Sugar cane bagasse	78.4% of carbohydrates and 21.6% of lignin.	Sugar cane bagasse was washed with hot water, dewaxed with toluene–ethanol, sieved to particles of 250–425 μm size, and dried at 50 °C for 24 h.	1-Allyl-3-methylimidazium chloride (AMIMCl)	Dispersed in AMIMCl, stirred at 110 °C for 5 h, phthalic anhydride (PA) and 4-methylaminopyridine added, stirred at 90 °C for 90 min, isolated by precipitation with ethanol, dissolved in DMF, casted onto mold	Sugar cane bagasse phthalates showed solubility in organic solvents, good film-forming properties, tensile strength of ∼ 33 MPa, Young’s modulus of ∼1680 ± 150 MPa and ∼3.25% elongation at break. Besides, they showed homogeneous architectural structure.	^45^
Sugar cane bagasse	46% of cellulose, 27% of hemicellulose, and 23% of lignin.	Sugar cane bagasse was washed and 20 to 40 mesh particles dewaxed by toluene/ethanol (2:1 v/v). The dewaxed sugar cane bagasse was oven-dried at 50 °C for 24 h and subjected to planetary ball-milled treatment for 4 h.	1-Ethyl-3-methylimidazolium acetate (EmimAc), 1-butyl-3-methylimidazolium chloride (BmimCl), and 1-allyl-3-methylimidazolium chloride (AmimCl)	Sugar cane bagasse (0.5 g) was dispersed into EmimAc (19.5 g) at RT. The mixture was placed in a 110 °C oil bath under N_2_ atmosphere for 5 h, vinyl acetate added, stirred, sugar cane bagasse esters obtained by precipitation, washed, vacuum-dried, films casted.	Good film forming properties, transparency, hydrophobicity, and excellent mechanical properties: tensile strength ∼20–55 MPa, Young modulus of ∼0.35–1.76 MPa and ∼2–60% of elongation at break.	^49^
Mulberry wood	49% of cellulose, 25% of hemicellulose, and 16% of lignin.	Mulberry wood was dried in sunlight, peeled and pulverized into 40 mesh size and oven-dried at 60 °C and further pulverized.	1-Butyl-3-methylimidazolium chloride (BMIMCl), 1-butyl-3-methylimidazolium acetate (BMIMAcO), and dimethyl sulfoxide (DMSO)	Mulberry wood was esterified in DMSO/IL, PA and DMAP added, modified wood precipitated, washed, films prepared by injection molding or solvent casting.	Highly translucent bioplastics containing cellulose I and cellulose II structures. Tensile strength in the 15–30 MPa range.	^47^
Poplar wood, Mulberry wood, and wheat straw	poplar wood: 45% of cellulose, 28% of hemicellulose, and 17% of lignin.	Materials were pulverized and ball-milled for 4 h. The size was reduced to 20 μm.	1-Methylimidazole (NMI)/ 4-(dimethylamino)pyridine (DMAP)/ phthalic anhydride (PA)	Lignocellulosic fibers esterified with PA in 1-methylimidazole, precipitated, extruded, and molded by injection into dumbbell-shaped specimens.	Effective esterification. Due to its softness, poplar wood esterified the most. The injection-molded samples were viscoelastic. Due to their high proportion of unaltered cellulosic components, which reinforced the specimens, mulberry wood-based polymers performed best in both flexural and tensile tests (tensile strength: mulberry wood ∼30–35 MPa, poplar wood ∼20–30 MPa, wheat straw ∼10–20 MPa)	^46^
	Mulberry wood: 49% of cellulose, 25% of hemicellulose, and 16% of lignin.					
	Wheat straw: 35% Cellulose, 34% Hemicellulose, and 16% lignin.					
Hybrid poplar wood chips	55.4% of cellulose, 12.5% of hemicellulose, and 27.7% of lignin.	Hybrid poplar wood biomass was dried at 40 °C, and material was then milled with a 40-mesh size.	1-Ethyl-3-methyl imidazolium acetate	Hybrid poplar wood biomass dissolved in IL solution, regenerated by spinning, filaments soaked, spun filaments collected, dried.	High mechanical tensile strength (∼105–122 MPa) and Young’s modulus varying from ∼30 to 38 MPa. The crystallinity of the fibers was 47–49%. Presence of strong intermolecular interactions and entangled structure.	^42^
Bagasse, cedar, and eucalyptus	bagasse: 41% of cellulose, 17% of hemicellulose, and 27% of lignin.	Particle size in the range 250–500 μm.	1-Ethyl-3-methylimidazolium methylphosphonate ([C2mim][(MeO)(H)PO2]) and 1-ethyl-3-methylimidazolium acetate ([C2mim]OAc)	Biomass was added into [C2mim] OAc, and heated at 120 °C for 2 h. Then, [C2mim] [(MeO) (H)PO_2_] was added and heated to 160 °C for 3 h under an Ar atmosphere. Water was added to the resulting samples, and the unreacted ionic liquids were removed by dialysis for 48 h. After filtration, the modified biomass was dried in vacuum and pressed at different temperatures (>140 °C).	The IL-modified biomass showed thermoplasticity and flame-retardant properties. Mechanical properties could not be achieved. After burning, the films showed foamed char layers and then they self-extinguished.	^63^
	cedar: 46% of cellulose, 15% of hemicellulose, and 36% of lignin.					
	eucalyptus: 42% of cellulose, 17% of hemicellulose, and 26% of lignin.					
Sugar cane bagasse	46% of cellulose, 27% of hemicellulose, and 23% of lignin.	Sugar cane bagasse was milled, dried, washed with dichloromethane, and dried.	1-Ethyl-3-methylimidazolium acetate/DMSO	Bagasse and EmimOAc were vacuum-dried at 80 °C for 24 h. After cleansing with Ar gas, DMSO was added and agitated at 110 °C for 16 h. Isopropenyl acetate was added to the cooled solution and stirred at 80 °C for 30 min after vinyl decanoate was added. Pouring the thick mixture into methanol, filtering, and washing. Dissolved residue was mixed in acetone for 16 h, centrifuged, and filtered. To get acetylated decanoylated polysaccharide, the acetone filtrate was concentrated and reprecipitated into methanol, then freeze-dried and vacuum-dried.	Long/short-chain mixed acyl groups in a 20:80 molar ratio (De/Ac) decreased the thermal processing temperature, allowing biomass injection-molding at 205 °C. The acetylated decanoylated polysaccharide, with a molecular weight of 1.5 × 10^6^ g/mol, showed remarkable mechanical capabilities, including 50 and 80 MPa tensile and flexural strengths.	^48^
Sugar cane bagasse	39% of cellulose, 28% of hemicellulose, and 28% of lignin	Milled, washed with dichloromethane, and dried.	1-Ethyl-3-methylimidazolium acetate (EmimOAc) and DMSO	Bagasse (6 wt %) was dissolved in EmimOAc and DMSO. After, vacuum-drying at 80 °C for 24 h produced a black-brown, homogeneous viscous solution. To obtain the vinyl esters, isopropenyl acetate (IPAc) (∼25 equiv/bagasse-[OH]) was added at 80 °C for 30 min. Short-chain acyls replaced bagasse’s OH groups after 30 min at 80 °C. Films were obtained by compression molding.	An excellent melt flowability at 180 °C, good mechanical properties (tensile strength: 35 ± 4 MPa and Young’s modulus: 1.6 ± 0.1 GPa).	^50^
Hybrid Pennisium grass, corn straw, wheat straw, and poplar sawdust	Hybrid Pennisium grass: 33.1% of cellulose, 39.8% of hemicellulose, and 6.4% of lignin; corn straw: 44.4% of cellulose, 42.2% of hemicellulose, and 3.2% of lignin; wheat straw: 45% of cellulose, 23.9% of hemicellulose, and 9.4% of lignin; poplar sawdust: 56.3% of cellulose, 22.6% of hemicellulose, and 16.8% of lignin	Biomass was treated in 2% NaOH at 60 °C for 0.5 h in order to remove a small amount of hemicellulose. After washing with water, the treated biomass powder aqueous dispersion (3%) was prepared	1-Allyl-3-methylimidazolium chloride (AmimCl), 1-butyl-3-methylimidazolium chloride (BmimCl), 1-butyl-3-methylimidazolium acetate (BmimAc), and 1-ethyl-3-methylimidazolium acetate (EmimAc)	The biomass powder was treated with 2% NaOH at 60 °C for 0.5 h. The treated biomass powder aqueous dispersion (3%) was made after washing. EmimAc was added to create a biomass/EmimAc aqueous dispersion under stirring. The biomass/EmimAc solids concentration was 20%. Biomass/EmimAc aqueous dispersion was put into a mold and dried to remove the water. The biomass/EmimAc mixture was hot-pressed for 5 min at 110 °C.	Tough and strong cellulose films and hydrogels were obtained. The solid content of cellulose/EmimAc was 20 wt %, the corresponding cellulose film exhibited an ultrahigh tensile strain of 57.7% and an excellent toughness of 81.76 MJ/m^3^. The cellulose hydrogel exhibited a tensile strength of 9.5 MPa and a high tensile strain of 171.4%.	^64^
Sugar cane bagasse	46% of cellulose, 27% of hemicellulose, and 23% of lignin.	Not reported	1-Ethyl-3-methylimidazolium acetate (EmimOAc)	One-pot two-step homogeneous transesterification of sugar cane bagasse in EmimOAc was conducted in the presence of vinyl ester (vinyl benzoate or vinyl hexanoate). The resulting mixtures were poured into distilled water, and the precipitate was vacuum-filtered and repeatedly washed with distilled water and vacuum-dried at 70 °C for 24 h and then turned into a film using a hot press machine.	The films made from bagasse had enhanced tensile strength, which was around 20 MPa, and exceptional ductility, as demonstrated by their high strain energy density ∼5 MJ m^–3^.	^65^
Sugar cane bagasse	39% of cellulose, 28% of hemicellulose, and 28% of lignin.	Pretreatment of bagasse is done by increasing the dissolution temperature from 110 to 140 °C to activate the weakly acidic proton at the C2 position of the Emim cation.	1-Ethyl-3-methylimidazolium acetate (EmimOAc)	Dried bagasse was dissolved in EmimOAc and DMSO. Partial decanoylation was achieved by adding vinyl decanoate and stirring at 80 °C. For peracetylation, excess isopropenyl acetate was added and mixed for 30 min. The mixture was put into acetone, filtered, and concentrated in a rotary evaporator. The amount of mixture was put into methanol to precipitate lignin. To get polysaccharide acetate decanoate, the residue was filtered, washed with methanol, freeze-dried for 2 days, then vacuum-dried at 70 °C for 24 h. The polysaccharide acetate decanoates were hot-pressed into films.	One-pot production of the enhanced polysaccharide-based plastics produce the bioplastic with higher thermal stability (Td–5% = 351 °C) and significant (*p* < 0.01) improvement of tensile properties (tensile strength = 38 MPa, elastic modulus = 1.4 GPa).	^66^

Overall, the strategy followed to obtain bioplastics from biomass using ILs involves biomass esterification by homogeneous^45,48,49^ or heterogeneous^46,47^ reactions with either phthalic anhydride (PA)^45–47^ or vinyl esters such as vinyl acetate,^48–50^ vinyl benzoate,^49^ vinyl laurate,^49,51^ vinyl decanoate,^48,50^ vinyl propionate,^50^ vinyl butyrate,^50^ vinyl hexanoate,^50^ vinyl octanoate,^50^ vinyl myristate,^50^ vinyl palmitate,^50^ and vinyl stearate.^50^ Vinyl esters are preferred over PA because the latter yield equivalent acids after reaction, leading to biomass degradation but not only. Phthalates, usually used as plasticizers in polymer materials and often found in most products that have contact with plastics, are recognized endocrine disruptors and teratogens, which is the reason why several countries are establishing restrictions and regulations for them.^52^ Likewise, in 2022 the FDA revoked authorizations for the food contact use of 23 phthalates, and the EFSA will re-evaluate the health risks from plasticizers such as phthalates and structurally similar substances.^53,54^

Although the use of ILs has demonstrated excellent capacity for biomass dissolution and subsequent obtaining of bioplastics, these compounds present several drawbacks. They are expensive and their preparation involves long reaction times at high temperatures, with the generation of a large amount of byproducts and residues that can become persistent pollutants in wastewater.^55^ In particular, imidazolium-based ILs are toxic to microorganisms such as bacteria, fungi, yeasts, plants, and animals, including humans, and are nonbiodegradable.^56–58^ In addition, they can be toxic to the environment since ILs can be potential contaminants of water and soil, especially during operational discharges or accidental releases.^58^ In this sense, recently, biocompatible ionic liquids (Bio-ILs) have been defined in the literature as ILs in which the anionic and cationic counterparts come from biobased renewable sources. For example, bio-ILs can be prepared with cholinium cations and organic acid anions coming, for example, from amino acids.^59^ Most cholinium-based ILs are considered biodegradable, biocompatible, and with low toxicity.^57^ However, up to now, they have not been employed for bioplastic preparation from the direct transformation of biomass. In view of the sustainability of the process and because of the fact the imidazolium-based ILs may represent a potential risk, are high priced, and are nonbiodegradable, their recycling either by washing or extracting with water or ethanol/methanol or using dialysis is highly important and must be considered also as part of the bioplastics preparation process when considering the LCA of these bioplastics.^60,61^

Lastly, ILs offer a slow rate of dissolution of biomass owing to the high viscosity of their solutions and often need pretreated biomass to improve their solubility.^62^ Even if their viscosity can be reduced with the addition of cosolvents like dimethyl sulfoxide (DMSO) or *N*,*N*-dimethylmethanamide (DMF), cellulose dissolution may still require time and temperature to dissolve which can reduce its molecular weight or the molecular weight of the other polymers present in the biomass.^41^ For instance, a recent investigation reported a significant reduction in the cellulose molecular weight with the increase in treatment time (1–24 h) and treatment temperatures (80 to 120 °C) when dissolved in the 1-ethyl-3-methylimidazolium acetate/DMSO solvent.^38^ These results added to the aforementioned disadvantages could seriously limit the applicability of this method for bioplastics production.

#### 3.1.2 Deep Eutectic Solvents

Deep eutectic solvents (DESs) represent an emerging category of environmentally friendly electrolyte substances that exhibit similar characteristics to ILs because of their similar high polarity and ability to solubilize a wide range of organic substances.^55^ However, DESs are different groups of substances. They are eutectic mixtures of two or more various hydrogen bond acceptors (HBAs) and hydrogen bond donors (HBDs) with examples in Figure 7. Thanks to their strong hydrogen bonding, their mixtures are liquid at room temperature, and even if they have a similar viscosity as ILs, this can be reduced by the addition of small quantities of water as a ternary component.^55^

**Figure 7:**
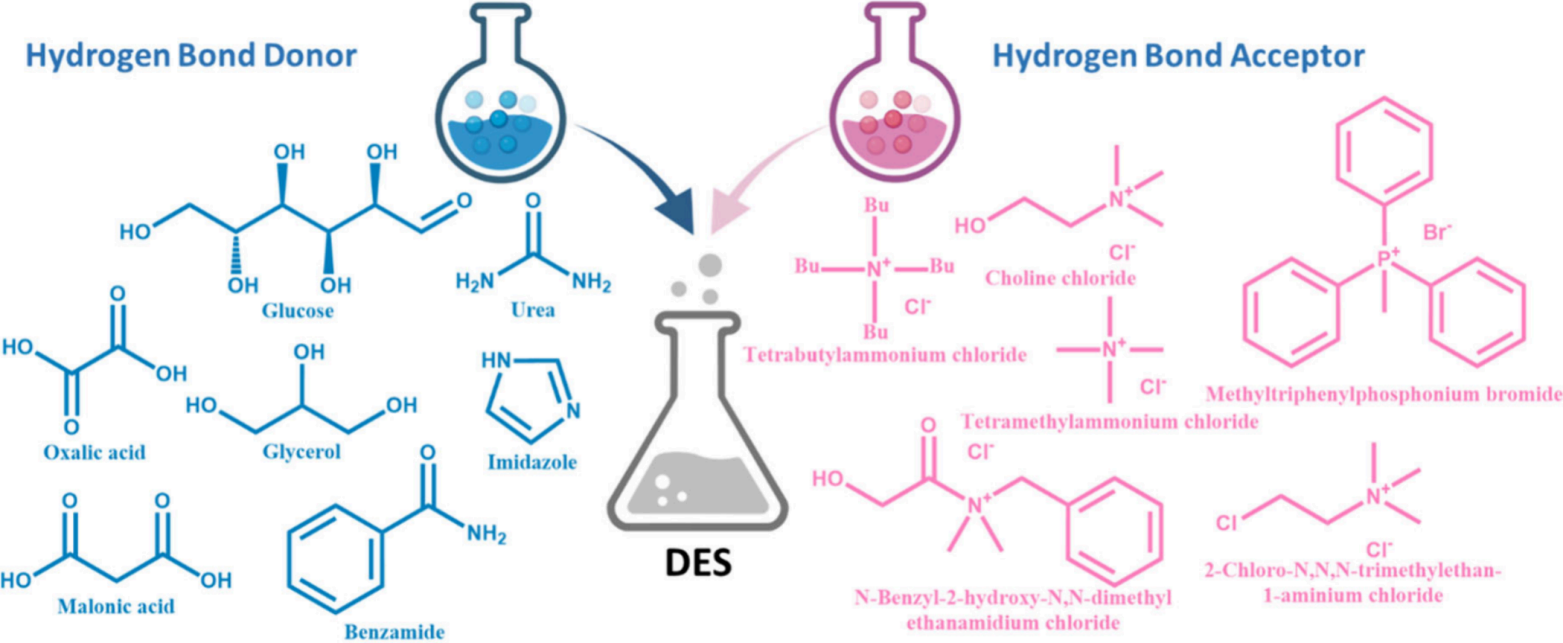
Examples of hydrogen bond donors (HBDs) and hydrogen bond acceptors (HBAs) commonly utilized in the preparation of DES.

In general, DESs are divided into four categories according to their composition (Figure 8). Type I consists of quaternary ammonium compounds combined with nonhydrated metal halides; type II consists of quaternary ammonium compounds combined with hydrated metal halides; type III includes quaternary ammonium salts and a hydrogen bond donor, such as urea, glycerol (a sweetener), or ethylene glycol (an preservative); and type IV includes metal chloride hydrate and hydrogen bond donor.

**Figure 8:**
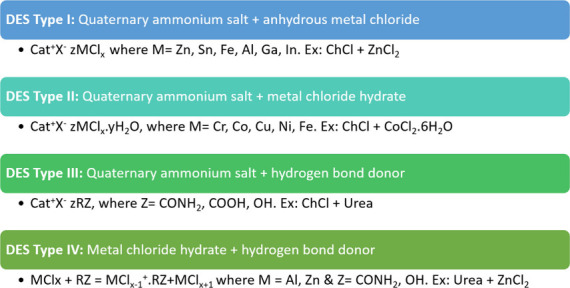
Classification of DES.

Among the different DES classifications, those of type III, which are commonly referred to as metal-free DESs, have been associated with a relatively smaller ecological footprint since they are less hazardous than their conventional counterparts. Furthermore, especially when derived from natural resources, they are designated as natural deep eutectic solvents (NADES).^67^

When compared to ILs, DESs stand out for the accessibility of their precursor materials, cost-effectiveness, inferior toxicity (particularly when sourced from specific biomass origins), and ease of preparation.^36^ DESs are obtained through simple and economical procedures involving the mixing of an HBD and a HBA at specific molar ratios and heating within the temperature range of 50 to 120 °C or alternatively through grinding in a mortar with a pestle or freeze-drying of their aqueous solutions.^55^ Notably, the production of DESs does not pose significant postpurification or disposal challenges, and they exhibit higher biodegradability and lower toxicity than ILs.^55^

Due to their notable properties, DESs have been used to prepare bioplastics directly from biomass. Most of the publications reporting on these preparations work on a laboratory scale using techniques such as solution casting and thermocompression. During these procedures, the DESs assist in the biomass dissolution and regeneration,^68^ but many publications deal with polymer and phytomolecule extraction^69^ and others with DES-assisted biomass blending, polymerization, or reactive processing methods,^70^ offering diverse approaches for environmentally friendly and sustainable bioplastic production.

Examples of the use of DESs for biomass deconstruction and bioplastics preparation are included in Table 2. In general, the process of biomass dissolution involves immersing the vegetable waste into a DES for a specified duration, which can vary depending on various factors, such as the type of DES, the nature of the biomass, and the desired outcome of the dissolution process. Generally, the immersion time can range from a few hours to several days. During this time, DES interacts with the biomass components, selectively dissolving certain polymers while leaving others unaffected. In the context of selective dissolution, cellulose typically remains insoluble. The selective nature of the dissolution is attributed to the specific interactions between DES and the polymers present in vegetable waste. Polymers such as lignin and hemicellulose are often more soluble, while cellulose, characterized by its strong inter- and intramolecular hydrogen bonding, tends to resist dissolution. As mentioned before, cellulose chains are densely packed in crystalline regions, further hindering solvent penetration and dissolution.^71^ This selectivity has been exploded in the literature to obtain specific fractions of biomass components, facilitating the extraction and utilization of target materials in various applications.^68^

**Table 2:** Summary of the Latest Published Works Reporting on the Dissolution of Biomass in DES for the Preparation of Bioplastics

Biowaste	Composition	Biomass pretreatment	Solvent	Procedure	Main results	References
Walnut shell	23.9% cellulose, 22.4% hemicellulose, 50.3% lignin, and 3.4% ash.	Walnut shell was crushed and sieved under 100-mesh size and then dried at 60 °C.	Choline chloride, toluenesulfonic acid monohydrate (p-TsOH), and ethylene glycol (molar ratios of 1:1:2)	Walnut shell and DES were mixed 1:10 wt % at 90 °C. After cooling to room temperature, 10 mL of EtOH was added and the mixture was centrifuged at 10,000 rpm. Multiwalled carbon nanotubes (MWCNTs) were added. A 10 mL dispersion was filtered and hot-pressed.	The film has high electrical conductivity (15.63 S/m) and tensile strength (63.86 MPa). Resistive responsiveness and flexibility allowed the composite film to monitor human physiological activity in real time as a biocompatible sensor.	^72^
Yoshino cherry (Plant periderm powder)	∼18% cellulose, ∼8% hemicellulose, and ∼46% lignin.	Plant periderm powder was sieved under 100-mesh size.	Choline chloride and oxalic acid dihydrate with a molar ratio of 1:1.	Plant periderm powder and DES (1:20 mass ratio) heated at 110 °C. Added distilled water (1:10 v/v), stirred 4 h, cooled overnight at 4 °C. Filtered, washed, and ultrasonicated. The slurries obtained were spread onto a hydrophobic surface to get a film.	Film with a tensile strength of 53.39 MPa and a fracture elongation of 93.7%.	^76^
					Room temperature recyclability by combining surface hydrophobicity and internal hydrophilicity. Reprocessing the plastic at room temperature with water droplets or hot-pressing and has no effect on mechanical performance.	
Poplar wood	46% cellulose, 30% hemicellulose, and 19% lignin.	Poplar wood was ground into powder and sieved under 60-mesh size.	Choline chloride and oxalic acid dihydrate at a molar ratio of 1:1.	Poplar wood and DES at a mass ratio of 1:15 were mixed and heated at 110 °C. Distilled water was added and stirred for 2 h. Then the slurry was filtered and washed using distilled water and spread on a hydrophobic substrate with a glass rod to obtain the lignocellulosic bioplastic film after evaporation of water at room temperature.	The bioplastic showed high mechanical strength, excellent water stability, ultraviolet-light resistance and improved thermal stability. Furthermore, the bioplastic could be easily recycled or safely biodegraded in soil, disappearing completely after three months.	^75^
Dead leaves of the red maple tree (*Acer rubrum*)	35% cellulose, 24% hemicellulose, and 30% lignin.	The leaves were thoroughly washed with pure water, laid out to dry at room temperature, and then ground into powders (≤300 μm).	Choline chloride and oxalic acid dihydrate at a molar ratio of 1:1.	A ∼6.5 wt % solution of leaves powder in the DES was prepared by heating to 100 °C for 30 min, and subsequent addition of deionized water and heating for another 2 h. The solid part was recovered by centrifugation and washed with distilled water. The slurry obtained was spread on a stainless steel plate and film was obtained after water evaporation at room temperature.	Films showed high performance in solar water evaporation, photocatalytic hydrogen production, and photocatalytic degradation of antibiotics. Furthermore, they acted as bioplastics with high mechanical strength, high-thermal stability, and showed complete biodegradation in soil after 40 days.	^77^
Poplar wood	35.85% cellulose, 40.90% hemicellulose, and 23.25% lignin.	Received as a powder and sieved by an 80 mesh screen.	Choline chloride and oxalic acid dihydrate at a molar ratio of 1:1.	Wood powder was mixed with distilled water, DES, or both, and ball-milled for definite times to obtain lignocellulosic slurry.	The bioplastics were more than 10 times harder than natural poplar wood, showed a water contact angle >60°, and a water absorption <20%.	^78^
				For the combination treatment by water and DES, the slurries were heated at 110 °C for 3 h.		
				All slurries were filtered and washed with distilled water, ultrasonic dispersed for 30 min, and centrifuged. Finally, the lignocellulosic slurries were put into molds and were dried at 60 °C for 6 h.		
Masson pine	50% cellulose, 11% hemicellulose, and 24% lignin.	Used as received	Choline chloride and lactic acid with a molar ratio of 1:10.	Masson pine lignocellulose was dissolved in the DES at different temperatures and times, and subsequently washed with deionized water until the pH was neutral. The samples were then dispersed and dissolved in AlCl_3_/ZnCl_2_ aqueous solution. After dissolution, lignocellulose was regenerated with ethanol and water and films were obtained by casting.	DES helped to increase the dissolution performance of the lignocellulose in the AlCl_3_/ZnCl_2_ aqueous system. Films had good ultraviolet blocking and hydrophobic properties. The tensile strength of the films reached more than 50 MPa. Films showed a 67% weight loss after 10 days biodegradation in soil.	^79^

A representative example for the bioplastics obtained with DES is given by the work of Haoxin et al.^72^ The authors used a DES prepared with choline chloride, toluenesulfonic acid monohydrate (*p*-TsOH), and ethylene glycol in molar ratios of 1:1:2 for the dissolution of walnut shell waste in combination with multiwalled carbon nanotubes for obtaining conductive films. As the authors explain, the use of DES aided in the lignin solubility and cellulose nanofribrillation and stabilized the dispersion of multiwalled carbon nanotubes. After that, the addition of water facilitated the regeneration of lignin, resulting in a slurry that was utilized to produce bioplastic films through compression molding. The resulting bioplastics exhibit desirable properties, such as high mechanical strength (128 MPa), improved water and thermal stability (357 °C), and biodegradability (5 months). This approach provides a sustainable alternative to traditional petrochemical-based plastics, contributing to the development of eco-friendly materials.

Another example is given by the work of Zhou et al. where the authors report the use of DES for the conversion of natural wood powder into bioplastic films by solution casting with a biomass valorization at a 74% yield.^73^ Choline chloride and oxalic acid DES with equal mole fraction were used to dissolve lignin and destroy hydrogen bonds between cellulose fibers and lignin. The DES treatment resulted in the formation of micro/nano-sized cellulose-lignin slurry with improved water resistance and hydrophobicity. The thermal stability and hardness of the all-lignocellulose-based bioplastics were also enhanced through the new assembly of cellulose and lignin molecules. TEM images showed the presence of regenerated lignin and nanocellulose in the samples, contributing to their superior hardness, as shown in Figure 9. Ball milling with DES not only eliminates lignin but also produces nanocellulose in smaller sizes, and loss in lignin content resulted in an increase in hydrogen bonding between fibers, thereby strengthening the interaction force among them. Moreover, the incorporation of nanosized and regenerated lignin further enhanced the interaction between fibers.

**Figure 9:**
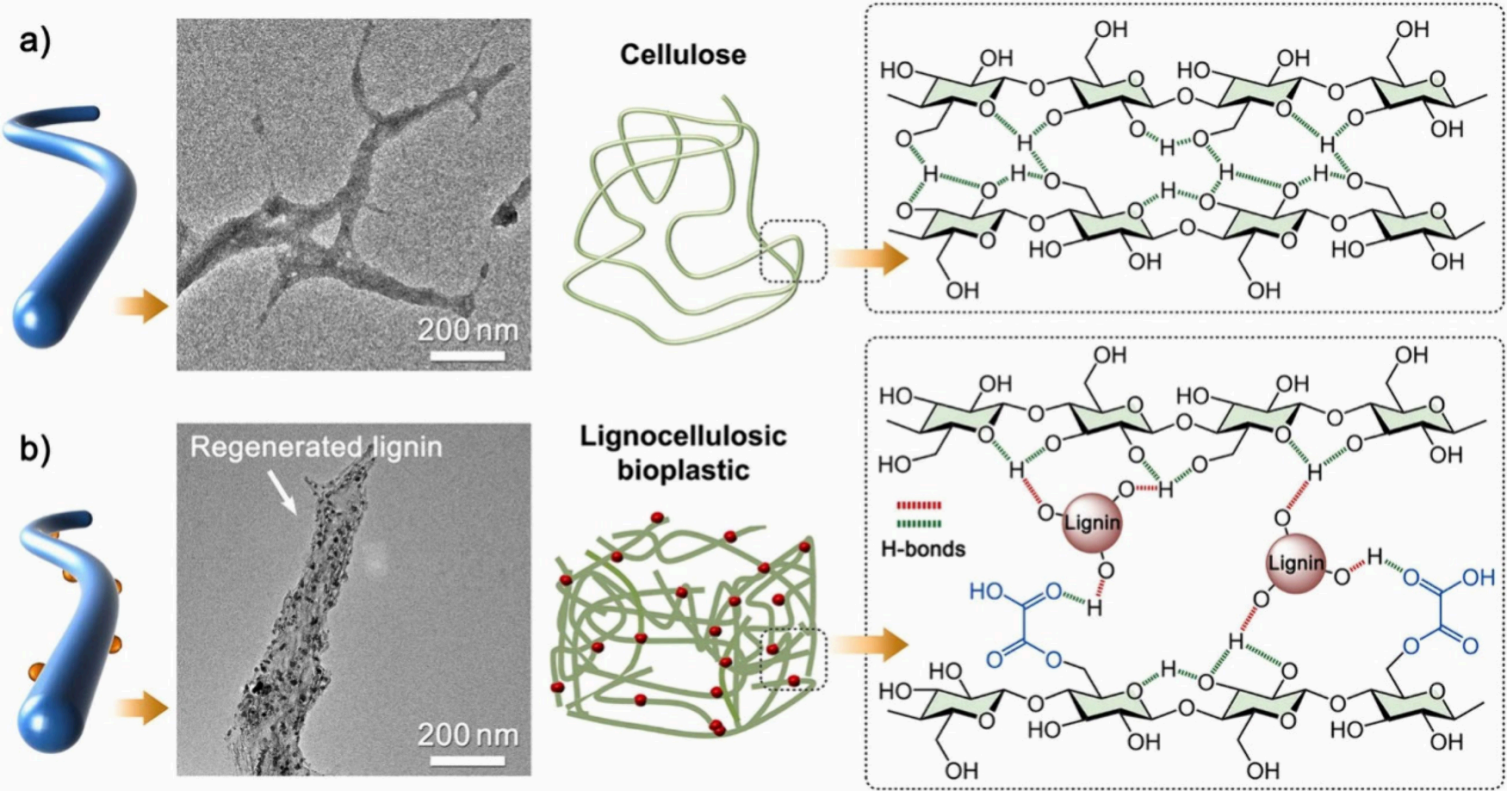
Structural linkages in cellulose and cellulose–lignin composites: (a) TEM image and schematic illustrating structural linkages between cellulose chains. (b) TEM image and schematic depicting the structural linkages between cellulose and regenerated lignin after treating poplar wood powder with ChCl:oxalic acid DES. Adapted with permission from Zhou et al.^74^ Copyright 2025 Elsevier.

The structure of the lignocellulosic biomass is distinguished by a rigid and interconnected arrangement, which is a result of the strong hydrogen bonding between cellulose fibers and lignin. The hydrogen bond donor (HBD) and hydrogen bond acceptor (HBA) constituents in DES have the ability to disrupt these hydrogen bonds, hence weakening the biomass matrix and facilitating the separation and dissolution of individual polymer chains. Choline chloride-based systems are more successful in dissolving lignin, whereas other DES variations are better at solubilizing hemicellulose or cellulose. DESs may selectively dissolve various biomass components. We can separate the useful parts of the biomass for further processing by this selective dissolving. In addition, DES enhances ionic interactions with polar functional groups, such as hydroxyl and carboxyl groups, thus disturbing the crystalline structure of cellulose and decreasing contacts between the components, ultimately enhancing solubility.

Similarly, Xia et al.^75^ have studied the use of ChCl:OxAcid 1:1 NaDES for the preparation of bioplastics directly from poplar wood. An in situ lignin regeneration technique is used to create the lignocellulosic bioplastic. In the first step, lignin and hemicellulose present in the wood powder are dissolved using a DES. After that, water is added to regenerate the lignin from the DES, followed by a filtration step, resulting in a cellulose–lignin slurry. This slurry is utilized in a straightforward casting method to produce lignocellulosic bioplastic sheets. As included in more detail in Table 2, the resulting bioplastic exhibits superior mechanical strength, water and thermal stability, recyclability, and biodegradability, making it a promising replacement for traditional plastics. Interestingly, the authors also evaluated the life-cycle assessment, which indicated low environmental impact, further reduced by utilizing renewable energy, highlighting the cost-effectiveness and scalability of the in situ lignin regeneration approach.

#### 3.1.3 Trifluoroacetic Acid

Another solvent that is widely used for the preparation of bioplastics through the dissolution of biomass is the TFA. TFA is a highly volatile organic acid, easily recyclable by distillation and miscible with other several organic solvents and water.^80^ The particularity of this solvent is that it allows the preparation of bioplastics in a simple and single step. After the biomass dissolution in the TFA, a process that can last several days, bioplastics can be obtained by casting and solvent evaporation. TFA vapor molecules can be recovered for the synthesis of new bioplastics, thus increasing the process efficiency and reducing its cost.^80^ Besides, this method does not require solvent exchange steps or postprocessing like other ionic solvents.^81^ Note that while TFA is an acid its primary role in reported applications has been as an anhydrous solvent for biomass dissolution—disrupting polymer networks—rather than as a hydrolytic reagent for controlled depolymerization.

A summary of the publications dealing with the transformation of biomass into bioplastics using TFA is included in Table 3. Bayer et al. were the first authors to report this method of preparation (Figure 10).^82^ In their work, the authors prepared bioplastics from wastes of parsley and spinach stems, rice hulls, and cocoa pod husks and claimed the obtaining of amorphous cellulose-based plastics in which many other natural elements present in these plants were plasticizing the amorphous cellulose.^82^

**Figure 10:**
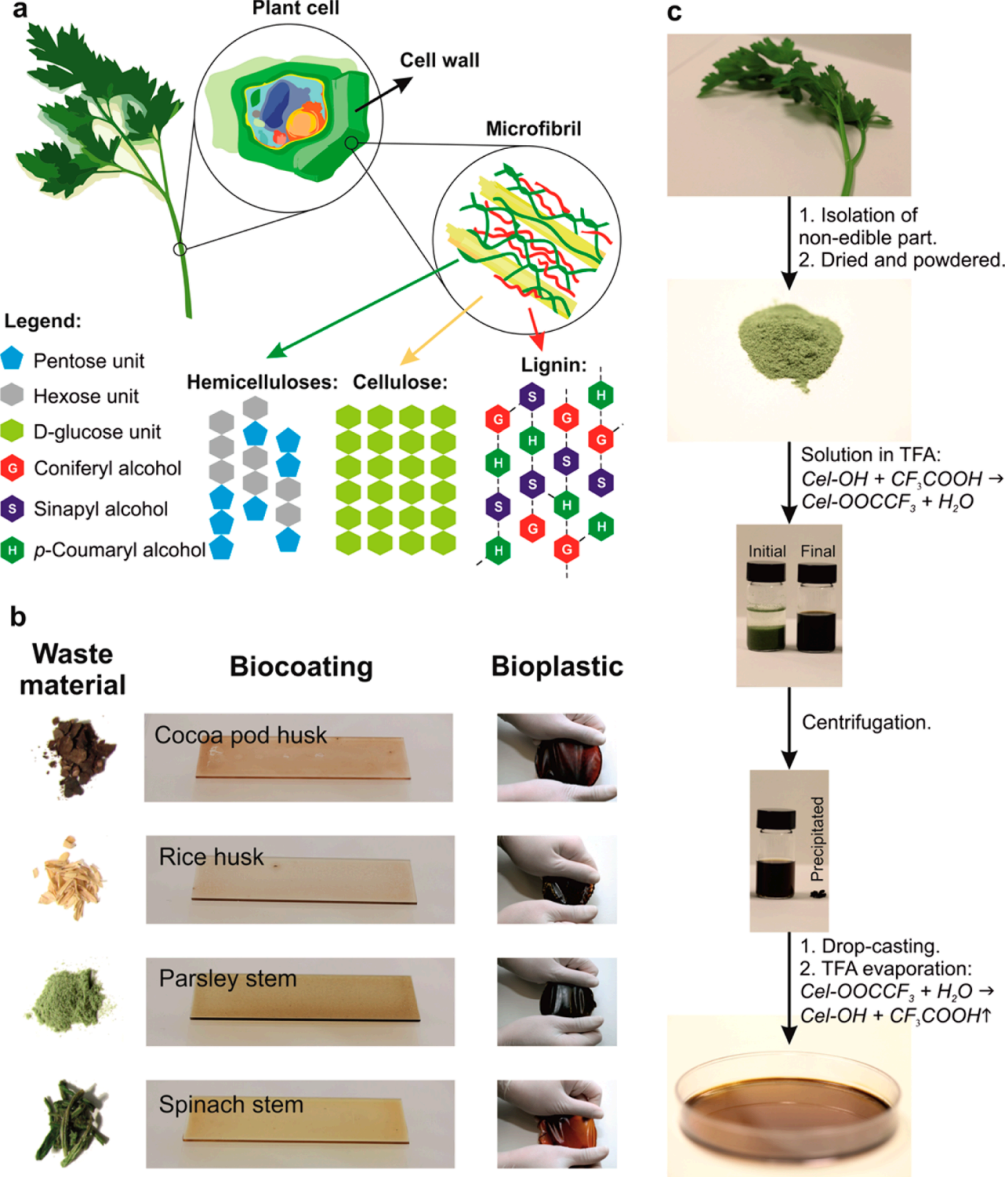
Transformation of plant waste into bioplastics by digestion in TFA. (a) Scheme of the main components (hemicellulose, cellulose, and lignin) present in the cell wall of inedible plant wastes and their distribution at different scales. (b) Different nonedible plant wastes. (c) Bioplastic production process. Reprinted with permission from Bayer et al.^82^ Copyright 2014 American Chemical Society.

**Table 3:** Summary of the Latest Published Works Reporting on the Dissolution of Biomass in TFA for the Preparation of Bioplastics

Vegetable waste	Composition	Biomass pretreatment	Solvent	Procedure	Main results	References
Parsley and spinach stems, rice hulls, and cocoa pod husks	Not reported	They were dried in an oven at 40 °C overnight even though they were provided already dehydrated.	TFA	Each material was dispersed in TFA at room temperature and was kept in a lab shaker for 29 days.	Depending on the plant species, biopolymers were found to display diverse mechanical properties ranging from brittle and rigid to soft and stretchable.	^82^
				Next, the obtained solutions were centrifuged in order to remove any residuals and the remaining clear solutions were cast in plastic Petri dishes for making free-standing films. The solutions were also spin coated on microscope glass slides.		
Rice straw (RS)	32–47% of cellulose, 19–27% of hemicellulose, and 5–24% of lignin	Extraction in Naviglio extractor.	TFA	Dried RS was ground into fine powder and sieved through 300 μm sieve. RS was mixed with TFA at a solid/liquid ratio of 1:20 (w/v). Then, it was maintained under magnetic stirring at room temperature for 3 days and subsequently casted.	The obtained solvent cast film was continuous, flawless, flexible and resistant to tearing. The bioplastic showed very good mechanical properties and promising dual-shape memory effect. Besides, it biodegraded within 105 days after buried in soil.	^80^
Parsley (*Petroselinum crispum*) stems	Rich in cellulose	Dried and ground to powder.	TFA	Hybrid nanofibers of fibroin (from *Bombyx mori* silkworm cocoons) and microcrystalline cellulose or cellulose-rich parsley waste were electrospun using trifluoroacetic acid (TFA) as a common solvent, without the need for additives or subsequent treatments.	Uniform distribution of the diverse components inside the nanofibers was observed.	^83^
					Noncytotoxic byproducts were formed due to the use of TFA, allowing the electrospun mats to be applied as scaffolds for promoting the proliferation of fibroblast cells.	
Red, green, and brown seaweeds (*Porphyayezoensis*, *Ulva lactuca*, and *Saccharina latissima*)	Not reported	Cellulose and seaweeds were dried in an oven at 40 °C overnight. After that, all the films were aged 7 days in TFA at ∼30 °C and shaking continuously.	TFA	Films were prepared by blending predetermined volumes of cellulose and seaweed solutions in order to obtain a final concentration of 75 wt % of seaweed in the composite biomaterials.	Mechanical properties were dependent on the seaweed’s origin. The bioplastics were fully biodegradable in seawater in one month. They presented antioxidant capacity and were not toxic, as demonstrated by biocompatibility and anti-inflammatory experiments.	^84^
Cocoa shells, orange peels, parsley stems, spinach leaves, cauliflower	Not reported	Dried and micronized vegetable powders were ground and then sieved with a 50 μm sieve.	TFA	Vegetables were mixed with anhydrous TFA and stirred gently at room temperature until the powder completely dissolved. For each vegetable, the disintegration period ranged from 48 h to 2 weeks.	The solvent-cast film that formed was precise and continuous. Biopolymers were discovered to exhibit a variety of mechanical qualities, ranging from brittle and inflexible to soft and flexible, depending on the species of plant. Furthermore, it degraded naturally in 30 days.	^85^

Other authors followed this pioneering work and reported similar methods of the preparation of bioplastics from other vegetable wastes. For example, Guzman-Puyol et al. reported the preparation of biocompatible nanofibers from silk and cellulose-rich parsley agro-waste.^83^ After that, in the work of Guzman-Puyol et al., the authors reported the preparation of bioplastics from the combination of commercial microcrystalline cellulose and three different seaweeds (red, green, and brown species).^84^ The authors stated that the complete dissolution of cellulose took 3 days under room conditions. However, algal solution required more days depending on the seaweed species: 7 days in the case of brown seaweed, 4 days for the red seaweed, and 5 days for the green seaweed (30 °C).^84^ TFA was also used to dissolve rice straw for the preparation of bioplastics in the work of Bilo et al.^80^ The authors reported that TFA was able to cosolubilize cellulose and other organic matter and that the laboratory scale production could be improved to an automated production processing.^80^ They also reported the presence of a brown color on the bioplastics and attributed it to the TFA ability to catalyze dehydration reactions on a number of organic compounds present in the biomass.^80^ Lately, Milionis et al. have used TFA for the dissolution of cellulose and subsequent casting on micropatterned Si wafers in order to obtain cellulose micropillar geometries.^81^ These authors also reported that for complete dissolution of cellulose a minimum of 3 days was required. Although TFA is highly volatile (it has a vapor pressure of 11 kPa at 20 °C), it was possible to find traces in the fabricated cellulose films. In fact, by the probe-electrospray ionization method they were able to determine that films contained up to 40 mg of bound TFA/kg of cellulose, but according to the authors, this concentration would not be enough to raise safety concerns.^81^ Among other disadvantages of the use of TFA, we should mention its toxicity, slow dissolution process taking even several weeks, and the necessity of anhydrous conditions.^85^

### 3.2 Hydrolysis of Biomass

Biomass hydrolysis is a process that involves breaking down complex organic materials, such as lignocellulosic biomass, into simpler compounds through the use of water or aqueous solutions. It serves as a fundamental step in biomass deconstruction, facilitating the disruption of cell walls and yielding viscous solutions of biopolymers. These solutions, upon solvent evaporation, transform into bioplastic composite films.

#### 3.2.1 Acid Hydrolysis

As an alternative method, hydrolysis of the plant cell wall in acidic media permits the redesign of cell-wall architectures in order to manufacture high-value products.

In the preparation of bioplastics, it is critical to prevent the excessive formation of monosaccharides or their degradation products by avoiding extreme conditions. Since macromolecules are required to produce plastic materials, mild conditions are preferred. The utilization of mild acid media to convert vegetable waste signifies an innovative, environmentally sustainable, and water-based procedure. By avoiding potential disadvantages associated with the utilization of organic solvents or ILs, such as TFA, this method guarantees enhanced compatibility with large-scale production processes.

As an illustration, Nishiwaki-Akine et al. documented the hydrolysis process of Japanese beech wood (*Fagus crenata*) with formic acid (Table 4).^91^ The acquired material exhibited flexibility and foldability without fracture, as illustrated in Figure 11.

**Figure 11:**
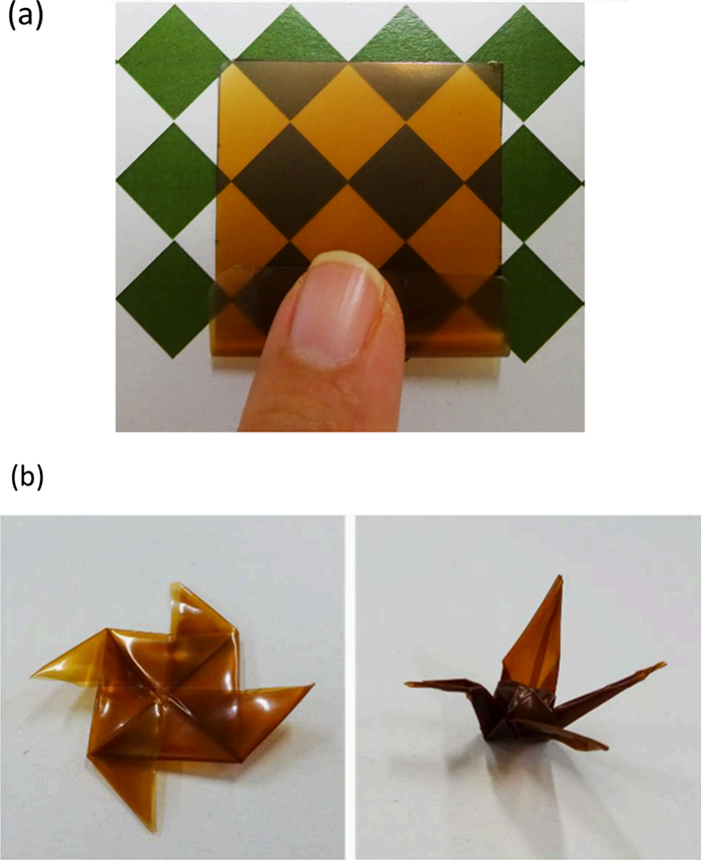
Photographs of (a) the woody film formed by evaporating formic acid after solution casting and (b) origami made by folding the woody films. Adapted from Nishiwaki-Akine et al.^86^ Copyright 2025 American Chemical Society.

**Table 4:** Summary of the Latest Published Works Reporting on the Hydrolysis of Biomass for the Preparation of Bioplastics

Vegetable waste	Composition	Biomass Pretreatment	Solvent	Procedure	Main results	References
*Fagus crenata* (Japanese beech) wood	Not reported	Ball-milled 0.5 h under nitrogen atmosphere and water cooling for 48 h.	99% formic acid	A mixture of the ball-milled wood and formic acid was stirred at room temperature for 5 days and films were obtained by casting onto polyethylene terephthalate (PET, Tetoron) substrate.	The films could be folded into various shapes when humid and they retained their shape and became harder when dried. Mechanical properties were relatively high, comparable to those of polystyrene, and films showed high biodegradability, similar to cellulose.	^86^
Carrot, parsley, radicchio, and cauliflower waste	Carrot: 61% cellulose, 28% pectin, 8% hemicellulose and 3% aliphatic polyesters. Parsley: 48% cellulose, 31% pectin, 15% hemicellulose and 6% aliphatic polyesters. Radicchio: 44% cellulose, 34% pectin, 4% hemicellulose and 18% aliphatic polyesters. Cauliflower: 46% cellulose, 24% pectin, 9% hemicellulose and 21% aliphatic polyesters.	The waste was received as dried powder and used without further purification or preprocessing.	5% (w/w) HCl	The vegetable wastes were dispersed in diluted aqueous HCl solution at 40 °C for 12 h. After dialysis for 72 h were casted onto Petri dishes.	Bioplastics were biodegradable, antioxidant and with similar mechanical properties of thermoplastic starch. They presented low migration of components in food simulant.	^94^
Cotton gin trash (CGT)	24–40% cellulose, 7–17% of hemicellulose and 18–29% of acid insoluble material containing lignin, condensed protein and lipids.	CGT without separating burrs, linters, leaves, stems, seeds and all other fine particles was chopped into coarse powder and passed through 0.2 mm size sieve.	99% formic acid	Mixtures of CGT powder and formic acid at different weight ratios were stirred at room temperature during 5 days. The mixture was then poured on a flat polystyrene dish to evaporate the formic acid (12 h).	Biodegradable films were obtained. Their crystallinity increased with increasing proportion of formic acid. They were thermally stable up to 200 °C without glass transition point and their TS was comparable to the one of low-density polyethylene.	^88^
Carrot pomace	52% cellulose, 43% pectin, 3% hemicellulose and 4% aliphatic polyesters.	The pomace was received as dried powder and used without further purification or preprocessing.	2 M formic acid	The carrot powder was dispersed in an aqueous solution of formic acid and stirred at 40 °C for 12 h. After that, the solutions were casted onto Petri dishes.	Materials developed were thermoplastic showing a glass transition temperature dependent on the water content of the Bioplastic film. Materials were able to be molded into complex forms when highly plasticized.	^87^
Carrot pomace	Not reported	The pomace was received as dried powder and used without further purification or preprocessing.	Anhydrous TFA, 2 M HCl and 2 M formic acid	The vegetable waste was dispersed in TFA (several days at room temperature), HCl or formic acid (12 h at 40 °C).	The bioplastics were more quickly biodegradable than pure crystalline cellulose. Films made with TFA were more transparent. Those obtained with HCl or formic acid were opaque because of the presence of cellulose crystals. Mechanical resistance was superior when using formic acid.	^85^
Apple pomace	38.9% Cellulose, 29.4% hemilcelluloses, 8.9% pectins, 23% lignin, and 2.9% starch	Apple pomace was either washed with water to remove free sugars or not washed and dried at 40 °C in a laboratory oven. The dried apple pomace was milled to a particle size of approximately 0.08 mm.	1% (w/v) citric acid	Apple pomace powder and glycerol were dissolved in citric acid solution. The mixture was heated to 70 °C under magnetic stirring at 560 rpm. The mixture was poured onto a nonsticky plate made of polytetrafluoroethylene for casting. The plates were dried at 40 °C in an oven.	Glycerol and natural sugars acted as plasticizers and improved cohesion among components. Authors prepared 3D objects by compression molding.	^89^
Spent tea leaves	Not reported	Received as a coarse, wet paste, it was dried under vacuum at 70 °C, ground in a coffee mill, and sifted to <355 mm particle size.	3% (w/v) citric acid	Tea waste powder was stirred in citric acid solution at 60 °C for 12 h and casted onto polystyrene Petri dishes.	Citric acid showed a 53.6% of esterification efficiency.	^90^
					Unreacted citric acid could act as hygroscopic plasticizer. Poor mechanical properties and hydrophobicity of the films surface due to films roughness.	
Potato peels (PoP)	30–85% starch, 24–65% fibers and 6–18% proteins.	Received as lyophilized powder and further ground to a size <250 μm.	1 M acetic acid	Hydrolysis was conducted at 40 °C for 16 h. After that, solutions were centrifuged to remove acid media and suspended in water. The suspensions were further heated at 80 °C for 1 h in presence of plasticizer. Films were obtained by casting and subsequent compression molding.	PoP films were homogeneous, transparent, and flexible. Polyglycerol plasticizer stand out for its good interaction with hydrolyzed PoP. It improved the thermal resistance and the mechanical properties of PoP-based films and presented better water barrier properties than the ones plasticized with glycerol.	^91^
Orange peels (OP) and spinach stems (SS)	OP: 39% cellulose, 31% pectin, 26% hemicellulose and 4% aliphatic polyesters. SS: 35% cellulose, 35% pectin, 16% hemicellulose and 15% aliphatic polyesters	Dried and powdered to a size <250 μm.	1 M acetic acid	Hydrolysis was conducted at different times and temperatures. For that, dried and powdered OP and SS were hydrolyzed in acetic acid at 30, 50, or 70 °C and for 6, 16, or 24 h under continuous stirring and casted onto Petri dishes.	Low temperatures (30 and 50 °C) and long times (16 and 24 h) were the most appropriate conditions for vegetable waste hydrolysis since higher temperatures (70 °C) had led to poor mechanical properties due to excessive degradation of constituent natural polymers. Bioplastics obtained presented rapid biodegradation in soil.	^92^
Avocado peels (AP) and seeds (AS)	AP: 38% cellulose, 37% hemicellulose, 10% lignin, 14% pectin, 1% polyesters. AS: 21% cellulose, 37% hemicellulose, 5% lignin, 13% pectin, 23% starch and 1% aliphatic polyesters.	The AP and AS were washed and stored at −18 °C. After that, they were dried in an oven at 40 °C for 48 h and finally ground to powder and sieved to a size inferior to 300 μm.	1 M acetic acid	Hydrolysis was carried out at 30 °C for 24 h, followed by 1 h at 80 °C in the presence of the plasticizer and subsequent blending with pectin polymer. Suspensions were casted onto Petri dishes.	The developed materials were suitable for their use in food packaging: appropriate mechanical and barrier properties, antioxidant capacity, biodegradability, and adequately low migration of components in Tenax food simulant.	^93^
Spinach stem (SS), peanut shell (PS)	SS: 35% cellulose, 16% hemicellulose, and 35% pectin, and PS: 39% cellulose, 15% hemicellulose, and 31% lignin.	SS and PS were dried and sieved under size 50 μm.	1 M NH_3_ or 1 M CH_3_COOH	Hydrolysis was carried out in 1 M NH_3_ or 1 M acetic acid aqueous solutions at 30 °C and for 8, 16, or 24 h under continuous stirring and casted on Teflon Petri dishes.	The materials prepared in 1 M NH_3_ exhibited superior fibril integration and dispersion in the polymeric matrix, as well as good mechanical and barrier qualities from SS bioplastics and PS composites.	^95^
Grass	50% cellulose, 20% hemicellulose and 18% lignin.	Dried and sieved under size 50 μm.	0.1 M; 0.5 M; 1 and 2 M NH_3_	Hydrolysis was carried out in NH_3_ aqueous solutions at 40 °C and for 8, 16, or 24 h under continuous stirring and casted on Teflon Petri dishes.	The developed bioplastics were suitable for sustainable packaging applications, exhibiting adequate mechanical strength, improved water resistance after mixing with ε-polylysine, notable antimicrobial and antioxidant properties, and reduced environmental impact, as confirmed by LCA and costing analyses.	^96^

According to the authors, the digesting process took 4–7 days at normal room temperature. This was determined by observing the transparency of the solution or suspension and the greatest rise in its viscosity, which occurred after 7 days. After that, the viscosity dropped owing to hydrolysis of the contents. In addition, they observed that the solution underwent a change in tone, becoming darker as the stirring duration increased. Softwoods are more difficult to dissolve in formic acid, as these authors also mentioned that the solubility of wood biomass in the acid depends on the amount of guaiacyl lignin (G). In fact, they reported that after several days at room temperature ball-milled cedar could not be dissolved in formic acid. Alternatively, Perotto et al. documented the breakdown of several nonlignocellulosic vegetable residues obtained from carrot, parsley, radicchio, and cauliflower using diluted HCl under gentle reaction conditions (as shown in Table 4).^87^ The bioplastic films developed had favorable mechanical characteristics, and the authors successfully shown that the hydrolyzed biomass may be combined with polyvinyl-alcohol (PVA), a polymer that dissolves in water, to enhance their ability to block oxygen. In addition, it was discovered that the gentle processing conditions did not modify the activity of certain molecules that are responsible for the antioxidant capabilities in vegetables. Nevertheless, the utilization of hydrochloric acid (HCl) in the hydrolysis procedure necessitated the subsequent elimination of the acidic medium using dialysis, a laborious and time-intensive operation.^87^ Subsequently, the authors also documented the process of breaking down carrot pomace using formic acid, which resulted in the same capacity for bending and shaping required for creating origami pieces.^87^ Furthermore, the authors assert that this acid does not generate any residues in vegetable waste-derived bioplastics, eliminating the need for dialysis.^87^

However, as reported by Simonutti et al. formic acid produces a large quantity of low molecular weight components that can easily migrate from the films during their hypothetic use for packaging of foods.^85^ These authors also compared different acids with TFA. They explained that when using TFA, which has the ability to dissolve cellulose crystals, amorphous and thus softer and stretchable bioplastics can be obtained. Instead, when formic acid is used, as it dissolves only pectin and hemicellulose, the material is more rigid. Something intermediate happens when using HCl. Since it can partially hydrolyze the cellulose, the amount of low molecular weight and amorphous components increases, creating a plastic and stretchable material.^85^ Further information in this regard was published in the work of Ahsanul Haque et al., who studied the hydrolysis of cotton gin trash (CGT) with formic acid (Figure 12), and found a correspondence between the Bioplastic crystallinity and the amount of formic acid used during the hydrolysis.^88^

**Figure 12:**
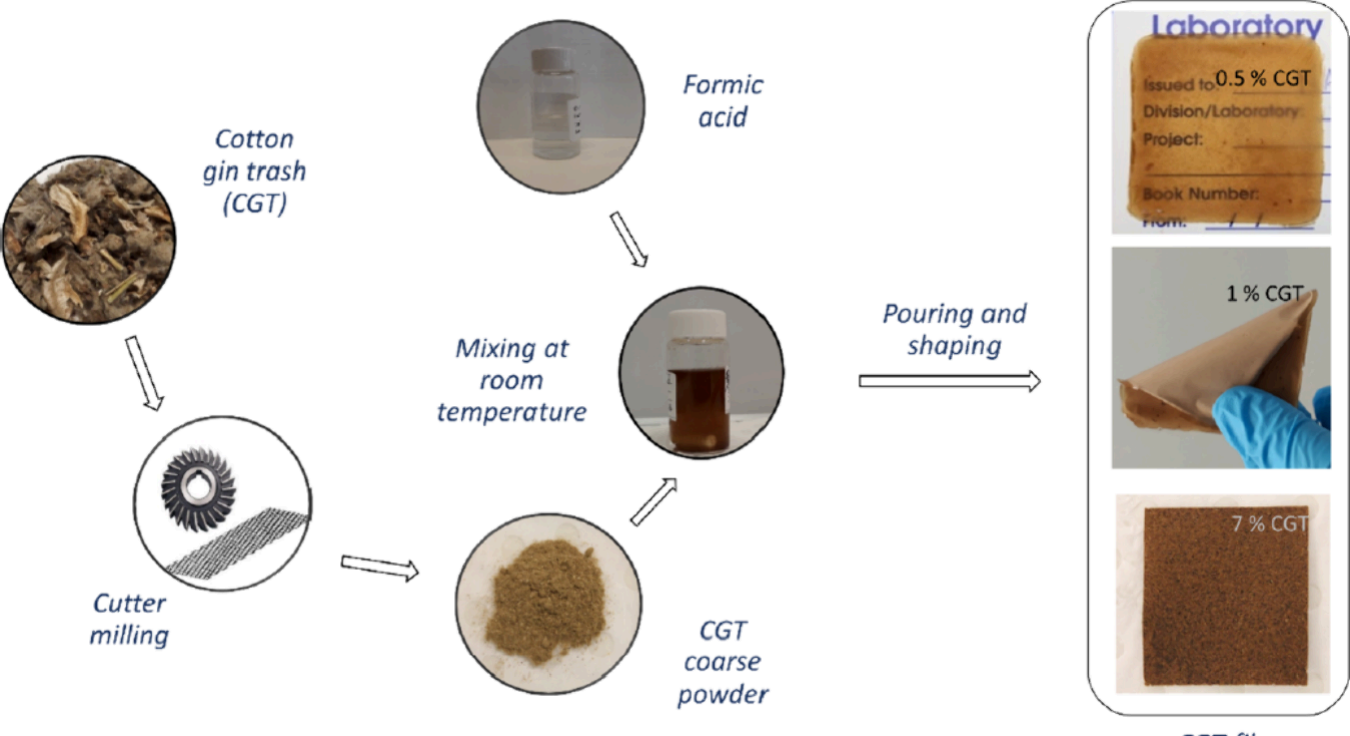
Schematic representation of the method followed by Ahsanul Haque et al.^88^ for the preparation of films from cotton gin trash.

Furthermore, citric acid was also proposed for hydrolysis of biomass and possible cross-linker.^89,90^ Although the results indicated a low efficiency of esterification, mechanical properties were improved when compared with the bioplastics without citric acid.^90^ Recently, Merino et al. used diluted solutions of acetic acid to hydrolyze potato peel waste, a starch-rich waste.^91^ In this work, due to the presence of starch, the hydrolysis alone was not sufficient to obtain flexible bioplastic films, and glycerol and polyglycerol compounds were evaluated as plasticizers, the latter showing the best mechanical properties and low migration of components in Tenax food simulant.^91^

More recently, Merino et al. have studied the effect of the hydrolysis time and temperature of different vegetable waste on the physiochemical properties of the obtained bioplastics.^92^ For that, spinach stems and orange peel waste were hydrolyzed in diluted acetic acid at 30, 50, or 70 °C and for 6, 16, or 24 h. Results indicated that low temperatures and longer times are the best conditions, leading to materials with the best mechanical properties. Drastic conditions, longer times, and high temperatures gave rise to the darkening of the bioplastics and reduced materials’ mechanical resistance due to the excessive pectin and hemicellulose degradation.^92^

Vegetable waste has also a significant portion of active molecules that are not removed or affected by the mild acid hydrolysis procedure. In the work of Perotto et al., radicchio bioplastics, rich in anthocyanin content, gave a similar antioxidant activity to the raw waste powder. Similarly, in the work of Merino et al., avocado wastes were processed by hydrolysis with acetic acid followed by plasticization with a polyglycerol and blending with pectin polymer.^93^ These films prepared with avocado peels, avocado seeds, or a combination of both wastes gave highly antioxidant properties with promising applications on food packaging. Therefore, this technique of processing vegetable waste does not affect the functionality of the biomolecules present in the vegetables and allows the acquisition of active bioplastics.

#### 3.2.3 Alkaline Hydrolysis

Alkaline hydrolysis is a versatile method for converting biomass into bioplastics by treating plant residues with alkaline solutions, such as sodium hydroxide or ammonia. The process typically begins with a mechanical pretreatment, such as milling and sieving, to facilitate solvent accessibility, followed by alkaline treatment under controlled conditions. This approach offers several advantages, including effective biomass deconstruction under mild conditions, cost-effectiveness, and reduced environmental impact compared to other chemical treatments.^96^

A notable study by Merino and Athanassiou demonstrated the *in situ* nanofibrillation of cellulose via alkaline hydrolysis using plant residues such as spinach stems (SS) and peanut shells (PS).^95^ The authors provided a comparative analysis of bioplastics produced through both acid and alkaline hydrolysis, evaluating their physicochemical, thermal, mechanical, and morphological properties. The results highlighted the superior performance of bioplastics produced via alkaline hydrolysis, which yielded improved mechanical strength and barrier properties relative to those derived from acid hydrolysis (see Table 4).

Bioplastics derived from spinach stems formed self-standing films, whereas hydrolyzed peanut shells yielded a powdery material. This powder was subsequently utilized as a reinforcing filler in thermoplastic starch (TPS)-based composite materials. The incorporation of hydrolyzed peanut shell fillers significantly enhanced the composite’s mechanical and barrier performance. These enhancements were attributed to efficient cellulose nanofibrillation, facilitated by the alkaline medium’s ability to partially hydrolyze pectin, hemicellulose, and lignin polymers (Figure 13).

**Figure 13:**
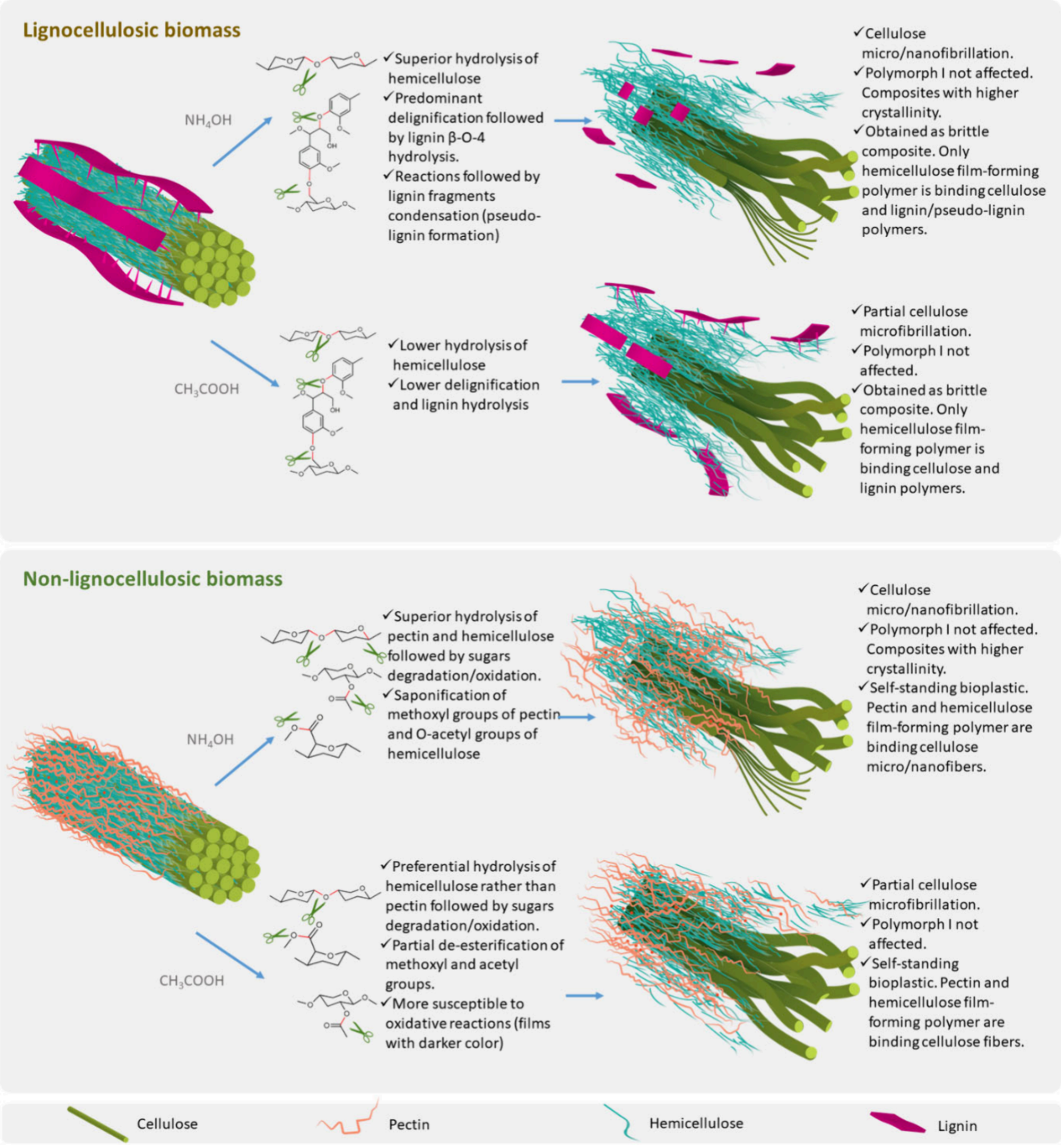
Summary of the mechanisms of acid and alkaline hydrolysis on lignocellulosic and nonlignocellulosic biomass. Adapted with permission from Merino and Athanassiou.^95^ Copyright 2025 Elsevier.

Building on this, a more recent study by Estrada-Sotomayor et al. explored the direct conversion of grass biomass into composite bioplastics via alkaline hydrolysis.^96^ The study focused on optimizing hydrolysis conditions, particularly ammonia concentration and reaction time, with the most effective conditions identified as 1 M NH_3_ and 24 h of reaction. The bioplastics were further functionalized through the incorporation of ε-polylysine, which conferred antimicrobial properties while also enhancing water resistance and mechanical strength (Young’s modulus ∼700 MPa; tensile strength ∼10 MPa). These multifunctional materials show strong potential for sustainable packaging applications. A life cycle assessment (LCA) of the process demonstrated substantial reductions in greenhouse gas emissions, energy consumption, and production costs compared to conventional plastic manufacturing.^96^

Together, these findings highlight alkaline hydrolysis as a promising strategy for transforming underutilized biomass into high-performance, multifunctional bioplastics, supporting the development of circular and sustainable material systems.

### 3.3 Thermomechanical Processing

Extrusion is a key example of thermomechanical processing. It involves a continuous mixing and kneading process widely used in the food and plastics industry due to its versatility, high productivity, low cost, continuous operation, and energy efficiency.^97,98^

The extrusion of vegetable products began in order to develop new foods, and it has been practiced for more than 50 years.^98^ The thermal and shear energy offered by the extrusion process allows the transformation of food raw materials in a structural, chemical, and nutritional way. For example, during extrusion, processes such as starch gelatinization, protein denaturation, lipid oxidation, vitamin degradation, and the formation of flavors, among others, occur.^98^

The extrusion of plant-based materials for the preparation of bioplastics could offer several advantages when compared with the methods based on the dissolution or hydrolysis of the biomass. Extrusion is an efficient and highly adaptable technique that typically does not require toxic chemicals or large amounts of water resources. Besides, it usually does not need additional separation or a drying process. In its most simple way, the chemical modification is realized through the combination of temperature, pressure, and kneading, and by varying these parameters different molecular structures and functional properties can be obtained, although it is still not well understood which cell wall components are the most susceptible to the thermomechanical stress.^99^

Some authors have studied the use of fruit and vegetable byproducts to enrich extruded snacks since they can provide minerals, vitamins, and dietary fiber.^98^ For example, Tonyali et al. processed wheat flour by extrusion with onionskin powder, rich in quercetin and fiber, and have found that the extrusion process increased the accessibility of quercetin and improved the antioxidant activity of the samples.^100^ Other authors have studied the effects of extrusion on physicochemical characteristics of cell walls of onion waste and demonstrated that extrusion increased the solubility of pectin and hemicelluloses polymers together with the swelling of the cell-wall material.^101^ Similarly, Schmid et al. concluded that the extrusion processing of apple pomace could disrupt the cell wall structure. These authors found that compared to the unprocessed raw material, extruded apple pomace showed an increase in its water solubility, water absorption, paste viscosity, and soluble dietary fiber. They also reported that at the harshest conditions low-molecular weight dietary fiber was obtained, from which arabinans (pectin neutral sugars side chain) were the most susceptible to the thermomechanical stress and xylans (type of hemicellulose) were more stable. Besides, these authors reported that the extrusion process is also responsible for the partial demethylation of pectins.^99^

Still, limited information is available on the thermomechanical processing of plant biomass for the preparation of bioplastics. As described before, examples involving the preparation of bioplastics by thermomechanical treatments may involve the use of ILs and organic solvents for the pretreatment of the biomass before its processing by extrusion or molding by injection.^102^

Some of these methods can be included in the category of reactive extrusion (REx) which is often considered a greener method of biomass modification. It combines heat and mass transport operations together with chemical reactions occurring during the processing inside the extruder. This technology is widely used to favor polymerization, grafting, cross-linking, and coupling reactions, in the materials science industry.^102^

For example, Vaidya et al. have used REx for the esterification of wood sander dust and the raw cellulose and hemicellulose extracted from it with different anhydrides: succinic anhydride (SA), maleic anhydride (MA), or dodecenyl succinic anhydride (DDSA).^103^ For that, the dried biomass was mixed with the anhydrides in various weight ratios using 3 wt. % of 1-methylimidazole as catalyst and extruded in a twin-screw extruded working at 300 rpm and temperatures ranging from 150 to 200 °C. The unreacted anhydride present on the extruded material was then extracted in a Soxhlet using a mixture of toluene:acetone:ethanol organic solvents, but still the catalyst was not possible to extract in these conditions.^103^ Moreover, although the authors reported a successful esterification of the biomass depending on the anhydride used, they still did not report the mechanical properties of the resulting materials.

Interestingly, pectin-rich biomass has been obtained as a bioplastic only by water plasticization and compression molding, using a hot press. The materials tested, carrot pomace, lemon peel, orange peel, spinach stems, and parsley stems, demonstrated the best mechanical properties when processed at 90 °C for 5 min under 5 tons since pectin polymer was susceptible to thermal degradation.^104^

An additional example of biomass transformation into bioplastics by thermomechanical processing is the study of Susanna et al.^105^ The authors indicated that whole potato peels may be efficiently utilized to create extruded films without necessitating a previous polymer extraction. The generated films had characteristics akin to those of conventional starch-based films, suggesting a feasible substitute for packaging materials.

## 4 Conclusions and Outlook

The valorization of plant biomass into bioplastics presents a promising, sustainable solution to the environmental challenges posed by conventional plastics. In this review, we have explored three advanced deconstruction strategies (dissolution, hydrolysis, and thermomechanical processing) that enable the selective breakdown and reassembly of biomass into functional materials. Each method offers distinct advantages and limitations, as summarized in Table 5.

**Table 5:** Comparative Summary of Biomass Deconstruction Strategies

Strategy	Target compounds	Frationation required	Advantages	Disadvantages	Future prospects
Dissolution (ILs, DES, TFA)	Lignin, pectin, hemicellulose, partially cellulose	Yes: For ILs and DES, solvent removal and precipitation of cellulose + lignin; hemicellulose remains dissolved and lost	High efficiency; selective deconstruction; customizable solvents	High cost; toxicity; complex solvent recovery	Development of biocompatible and recyclable solvents; enhancing DES dissolution efficiency
Hydrolysis (acid, alkaline)	Mainly hemicellulose and pectin; partially cellulose (severity-dependent)	No	Targeted polymer breakdown; preserves key component functionality	Degradation risk; requires solvent neutralization or recovery	Mild acid/alkaline conditions; integration with downstream processing
Thermomechanical processing	Mostly hemicellulose and pectin. REx can target cellulose, hemicellulose, pectin, lignin.	No	Minimal chemical use; energy-efficient; versatile	Limited polymer selectivity; potential polymer degradation	Precision control of parameters; integration with solvent-based or chemical methods

Dissolution techniques, especially those using ILs and DES, are highly effective for selective biomass deconstruction. NADES systems, in particular, offer low cost, biodegradability, and simple preparation; however, challenges related to toxicity, solvent cost, and recovery (especially for ILs) must be overcome to improve scalability. Compared to ILs, DES generally dissolves biomass less efficiently, and TFA, while simpler, suffers from toxicity, volatility, and slower processing.

Hydrolysis, whether acidic or alkaline, enables the controlled breakdown of polymers like hemicellulose, pectin, and lignin, preserving much of their polymer functionality for downstream bioplastic applications. However, the process generates acidic or alkaline byproducts that must be neutralized or recovered, and harsh conditions can lead to polymer degradation. Alkaline hydrolysis is especially effective for lignin separation, making it valuable for lignocellulosic biomass transformation, although its water consumption can be seen as a disadvantage.

Thermomechanical processing, such as extrusion, offers a greener route by minimizing or eliminating chemical inputs. This strategy excels in energy efficiency and operational simplicity, but it lacks the polymer selectivity of solvent-based approaches and requires careful control to prevent degradation.

Notably, while these studies detail each method’s qualitative strengths, there remains a paucity of systematic, quantitative data (yields, component purities, and reductions in molecular weight or degree of polymerization) across dissolution, hydrolysis, and thermomechanical routes. Addressing this gap would enable direct benchmarking and inform the design of optimized hybrid workflows. Looking forward, the integration of these strategies is key to maximizing efficiency, sustainability, and economic viability. For instance, combining solvent systems with thermomechanical processes or coupling mild hydrolysis with downstream extrusion could overcome the limitations of individual methods. To truly benchmark these hybrid approaches, future work must include standardized analytics and reporting protocols that capture yield and degree-of-polymerization changes. Furthermore, designing pilot-scale systems will be crucial for evaluating environmental impacts and process feasibility. Exploring underutilized feedstocks—such as marine algae or invasive plants—and refining preprocessing techniques can unlock new biomass sources. Ultimately, a multidisciplinary effort spanning chemistry, materials science, environmental engineering, and economics will be essential to overcome challenges related to scalability, solvent toxicity, and polymer degradation and to usher in truly sustainable, biomass-derived plastics.

## Data Availability

No new data were generated or analyzed in this review. Data supporting the conclusions of this article are available in the referenced publications and can be accessed online.
